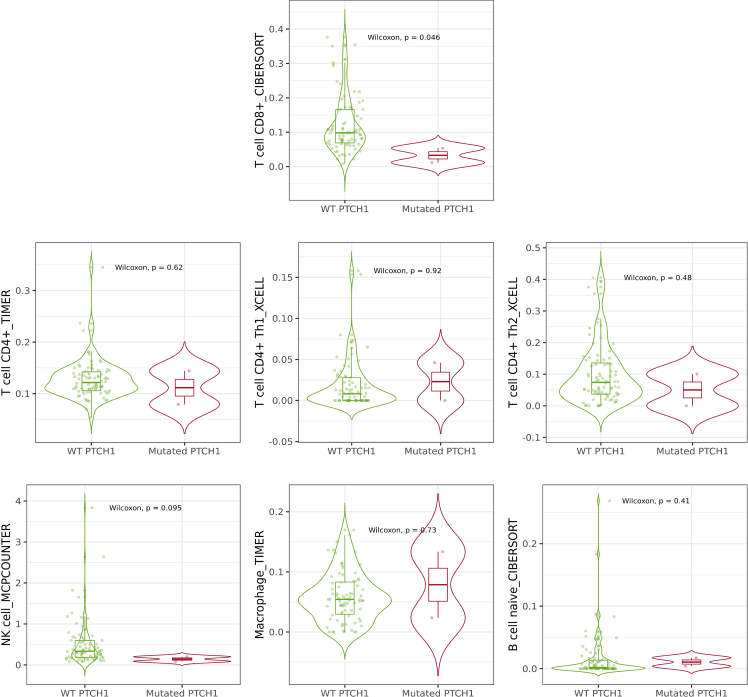# In-depth profiling of tumor tissue derived from malignant pleural mesothelioma patients identifies potential biomarkers predicting response to immune-checkpoint inhibitor therapy

**DOI:** 10.1016/j.gendis.2023.101189

**Published:** 2023-12-03

**Authors:** Dmitrii Shek, Bo Gao, Hema Mahajan, Adnan Nagrial, Matteo S. Carlino, Fabio Luciani, Scott A. Read, Golo Ahlenstiel

**Affiliations:** a Blacktown Clinical School, Western Sydney University, Sydney, NSW 2148, Australia; b Westmead Institute for Medical Research, Sydney, NSW 2145, Australia; c Blacktown Mt Druitt Hospital, Sydney, NSW 2148, Australia; d Westmead Hospital, Sydney, NSW 2145, Australia; e Westmead Clinical School, University of Sydney, Sydney, NSW 2145, Australia; f Institute of Clinical Pathology and Medical Research, Sydney, NSW 2145, Australia; g Melanoma Institute Australia, Sydney, NSW 2065, Australia; h School of Medical Sciences, University of New South Wales, Sydney, NSW 2052, Australia; i Garvan Institute for Medical Research, Sydney, NSW 2010, Australia

Malignant pleural mesothelioma (MPM) is a rare and aggressive cancer with low survival probability as it is generally diagnosed at later stages.^1^ Using a combination of immune-checkpoint inhibitors (ICIs) ipilimumab (IPI) and nivolumab (NIVO) as a first-line treatment for unresectable MPM, the CheckMate 743 trial reported higher overall survival and prolonged duration of response compared with traditional chemotherapy.^1^ This combination has recently been approved as a new first-line standard of care for patients with advanced MPM, although the incidence of immune-related adverse events reached 80 %, with 31% of patients experiencing grade 3-4 immune-related adverse events (irAEs).^1^ The unpredictable nature and severity of immune-related adverse events emphasize a critical need to conduct in-depth translational studies to characterize the tumor microenvironment and systemic immune response that contribute to the efficacy and safety outcomes of ICI therapy.

For this pilot study, we recruited four males with metastatic MPM of Caucasian origin treated with IPI (1 mg/kg every 6 weeks) and NIVO (3 mg/kg every 2 weeks). The median age was 74 years old, and all patients had prior asbestos exposure. Patients were encoded as A1, B1, C1, and D1, and their baseline clinical characteristics are available in Table S1. The 3 months computed tomography (CT) scans measured using RECIST 1.1 guidelines showed that A1 and C1 had a complete response (CR) and stable disease (SD) respectively and B1 and D1 had disease progression (DP). The FFPE blocks from the diagnostic tumor biopsy (A1, B1, and C1) and from the metastasis to the temporal lobe (D1) were collected and used for Visium (Fig. S1) spatial gene expression profiling (10x Genomics) and SENTIS + Cancer discovery mutational panel (BGI Genomics). Detailed methodologies are available in Supplementary Materials. This pilot study aimed to investigate transcriptomic variations within tumor-infiltrating immune cells, particularly T cells and mutational profiles of MPM tissues focusing on their potential association with the response to ICI therapy.

To determine the location of tumor-infiltrating T cells within Visium spatially profiled FFPE tissues, we utilized an *ImSig* package in R as we have shown before.^2^ Tissues A1 and C1 possessed a greater number of spatial spots assigned to T cells as compared to non-responders [*n* = 250 (5.6% from total spatial spots covered by tissues A1 and C1) and *n* = 137 (2.9% from total spatial spots covered by tissues B1 and D1), respectively] (Fig. 1A). Using the *BayesSpace* package^2^ to increase the spatial resolution 5-to-6-fold, we were next able to quantify cell interactions at an almost single-cell resolution. We established that the percentage of tumor spots directly surrounded by T cells was A1 = 3.8%, B1 = 0.61%, C1 = 4.75%, and D1 = 6.76% with no significant difference between responders and non-responders (*P* = 0.85).

**Figure 1:**
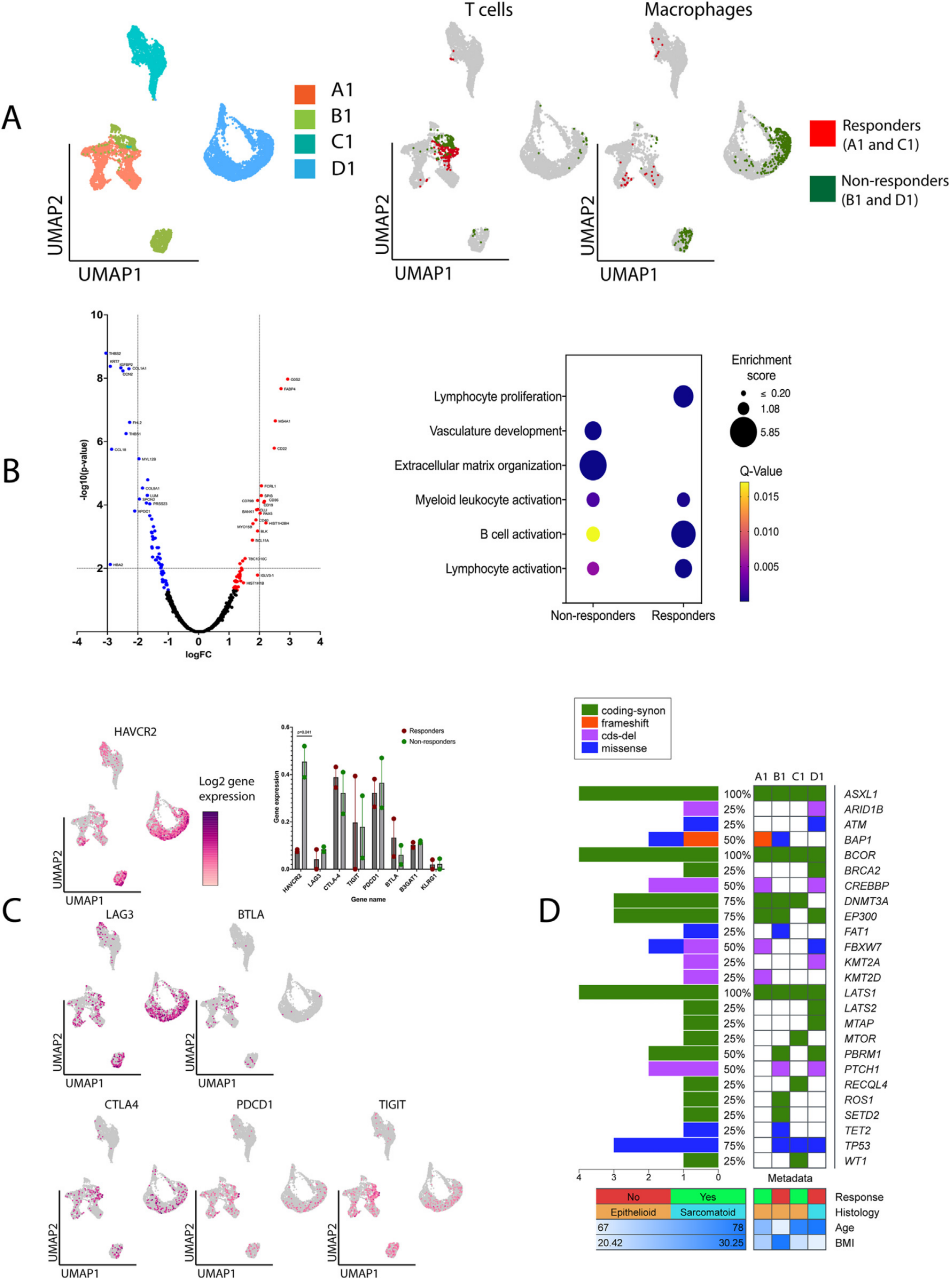
Multiomic signatures of malignant pleural mesothelioma associated with the immunotherapy outcomes. **(A)** Top left UMAP represents the stochastic location of spatial spots examined through Visium spatial analysis and assigned to each of the 4 examined tissues (A1, B1, C1, and D1). Two other UMAPs represent the location of spatial spots assigned to tumor-infiltrating T cells and macrophages identified through the *ImSig* algorithm in responders (A1 and C1) and non-responders (B1 and D1). **(B)** The volcano plot represents differentially expressed genes between T cells of responders and non-responders. Genes significantly downregulated and upregulated in responders are highlighted in blue and red respectively. Plot to the right represents Gene Ontology terms enriched by significantly upregulated genes in the T cells of non-responders and responders. **(C)** UMAPs represent the distribution of immune-checkpoint inhibitor genes. The expression of checkpoint inhibitory gene *HAVCR2* was significantly higher in tumor-infiltrating macrophages of non-responders (*P* = 0.041). **(D)** Landscape visualization of mutational analysis. The main oncoplot visualizes the genomic alterations that occurred in studied genes related to MPM. Additional (bottom) panels represent the clinical response to ICI therapy (categorical), histological type of MPM (categorical), age (numerical), and pre-treatment body mass index (numerical).

We next conducted a differential gene expression analysis on T cell harboring spots that identified 122 dysregulated genes; 70 and 52 were significantly upregulated in non-responders and responders, respectively. Annotation of these gene sets based on response status identified unique biological processes that have the potential to contribute to therapy response (Fig. 1B and Table S2, 3). Responders showed a stronger enrichment of T cell genes responsible for lymphocyte proliferation and myeloid and B cell activation, indicating that responder T cells are better interacting with activated leukocyte populations to stimulate their proliferation proximal to the tumor site. In contrast, non-responders demonstrated activation of pathways associated with extracellular matrix organization and vasculature development. Dysregulation of extracellular matrix production increases remodeling of the tumor microenvironment^3^ and has been shown to mediate more aggressive cancer behavior by enhancing stromal stiffness, altering cell signaling, and impacting drug delivery to ultimately shield tumor cells from therapies such as ICIs.^3^ Furthermore, neo-angiogenesis supports cancer growth as well as creating new physical barriers preventing effective infiltration of cytotoxic T cells.

We next selectively examined the expression of immune-checkpoint inhibitor genes (*HAVCR2*, *LAG-3*, *BTLA*, *CTLA-4*, *PDCD1*, and *TIGIT*) on cancer, T cells, and major antigen-presenting cells (macrophages). Interestingly, no differences among cancer cell or T cell spots were identified stratified by therapy response. Upon examination of macrophage-resident spots, the expression of *HAVCR2* was found to be higher in non-responders (Fig. 1C). *HAVCR2* encodes an inhibitory checkpoint molecule TIM-3 which is involved in the regulation of macrophage polarization.^4^ Jiang *et al*. demonstrated that up-regulation of TIM-3 results in M2 (anti-inflammatory) polarization of macrophages positively correlated with tumor growth.^4^ Moreover, the abundance of M2 macrophages in MPM pleural effusions is associated with lower infiltration of T cells and T cell proliferation *in vitro* supporting its immunosuppressive function.

TIMER 2.0 was used to determine if the expression of *HAVCR2* was associated with overall survival in chemotherapy-treated MPM patients. Interestingly, the Cox proportional hazard model in TIMER 2.0 determined that high expression of *HAVCR2* combined with a higher number of infiltrating macrophages is associated with lower overall survival in MPM as compared with a lower number of infiltrating macrophages (Fig. S2). Further investigations are warranted to validate these preliminary transcriptomic findings and to unravel specific genetic signatures of tumor resistance to IPI + NIVO therapy.

Finally, we aimed to determine a tissue mutational landscape using the SENTIS + panel (BGI Genomics). Our analysis established that gene *PTCH1* was exclusively mutated (deletion of triplet GCC on 9q22.32) in tumor tissues of non-responders (Fig. 1D). Large deletions (>4.5 Mb) within the identified locus are associated with rare nevoid basal cell carcinoma.^5^ Nonetheless, it is currently unknown if the identified microdeletion can impact MPM progression and resistance to ICI therapy, and further studies are warranted. Next, we investigated the copy number variations across examined tissues. We did not establish any pattern for copy number variation gain or loss related to the therapy response. However, both tissues B1 and D1 showed copy number gain (both germline and somatic) of the gene *HLA-B*. In contrast, neither copy number variations nor other type of mutations in *HLA-B* were detected in A1 and C1. Analysis in TIMER 2.0 established that *PTCH1* mutations are significantly associated with lower infiltration of CD8^+^ T cells in MPM with no association with CD4^+^ T cells, B cells, NK cells, and macrophages (Fig. S3). The identified mutation in *PTCH1* raises intriguing questions regarding the implication of pre-existing somatic and germline mutations on MPM resistance to IPI + NIVO therapy. Further in-depth investigations are imperative to validate the precise mechanisms underlying these pilot findings.

In summary, this pilot analysis provides valuable insights into the tumor-based molecular signatures associated with the response to IPI + NIVO therapy in MPM patients. While the study has several strengths, particularly the implementation of high-throughput methods such as Visium spatial analysis, it is also crucial to highlight some limitations. Notably, the small number of samples is a major limitation that may compromise the external validity of the findings. Moreover, the absence of a confirmation cohort limits the opportunity to validate the results and account for potentially contributing confounding factors such as tissue collection site, method, and FFPE preservation technique. Nevertheless, this study provides important insights into spatial and cell-specific transcriptomic differences between therapy responders and non-responders in a real-world cohort of patients, thus offering a significant step needed to identify biomarkers of ICI therapy. The ongoing recruitment in NCT04631731 holds promise for the successful validation of preliminary data thus providing new perspectives on establishing risk factors of ICI therapy in the near future.

## Ethics declaration

Research sample collection and analysis were conducted as part of the NCT04631731 study (ICEMELT), which has been approved by the Western Sydney Local Health District (WSLHD) Human Research Ethics Committee (Ethics reference number: 2020/ETH02285). Each recruited patient has provided written informed consent for participating in ICEMELT (NCT04631731) study.

## Author contributions

Bo Gao and Golo Ahlenstiel designed and supervised the study. Bo Gao, Adnan Nagrial, and Matteo Carlino provided clinical care. Hema Mahajan completed the pathological annotation of the examined tissues. Dmitrii Shek, Scott Read, and Golo Ahlenstiel completed the data analysis. Fabio Luciani contributed to transcriptomic data analysis. Dmitrii Shek, Scott Read, and Golo Ahlenstiel wrote the manuscript. All authors contributed to the critical revision of the manuscript and approved its final version for publication.

## Conflict of interests

The authors declare no conflict of interests.

## Funding

The NCT04631731 is funded by (i) Western Sydney Local Health District Research and Education Grant 2021, (ii) Bristol Myers Squibb (CA209-6KR), and (iii) BGI ANZ Genetic Service Grant 2021.

## Data availability

The data is available upon request to a corresponding author.

## Figures and Tables

**figs1:**
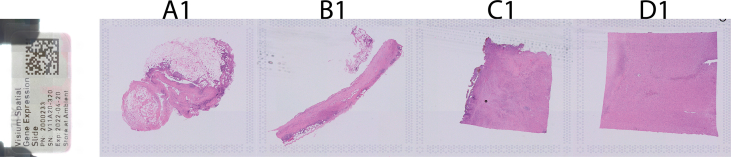


**figs2:**
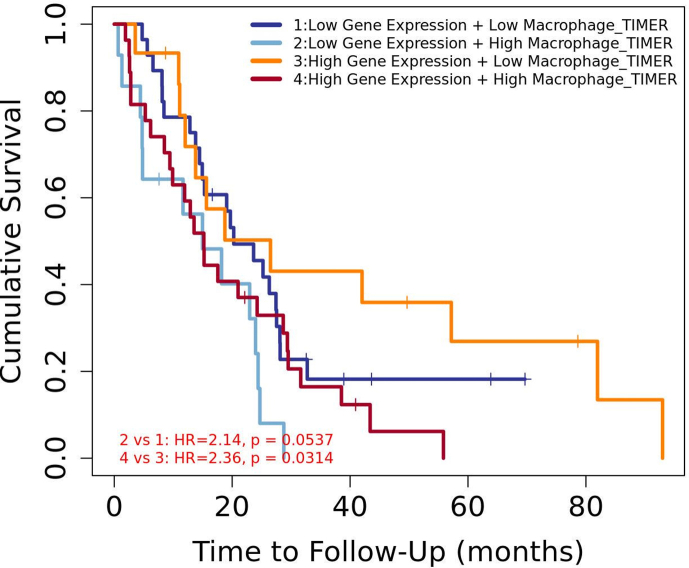


**figs3:**