# Supplementary material for: In-depth profiling of tumor tissue derived from malignant pleural mesothelioma patients identifies potential biomarkers predicting response to immune-checkpoint inhibitor therapy
Source: Genes Dis. 2023 Dec 3;11(6):101189. doi: 10.1016/j.gendis.2023.101189 (PMC11298826; doi:10.1016/j.gendis.2023.101189)
Supplement: Multimedia component 1 [file mmc1.docx]

**SUPPLEMENTARY DATA**

**Methods of VISIUM spatial transcriptomics (10x Genomics).**

We strictly followed the protocol provided by 10x Genomics without deviations. Each tissue section was placed within one of four 6.5 x 6.5mm capture regions of the Visium slide. Sections were left overnight to dry and incubated the following day in a 60-degree fan-forced oven for 2 hours and stained with H&E. H&E-stained slides were coated with 85% Glycerol and a coverslip and imaged with the Olympus VS120 Slide Scanner at 40x lens magnification. After imaging, glycerol was washed off by immersing the Visium slide into the beaker containing 800 mL of Milli-Q water. Samples were decrosslinked using recommended TE buffer (pH 9.0) before human transcriptome probes covering 18,000 genes were ligated to target mRNA for capture and library construction according the the Visium Spatial Gene Expression for FFPE protocol with no deviation. Sequencing was performed on a Novaseq SP flow cell (Illumina) with 50bp paired end read length. Approximately 100M paired reads per acquired for each sample and Fastq files were created using the bcl2fastq 2.20.0.422 pipeline. Gene expression counts were generated from fastq files using space ranger V1.3 with refdata-gex-GRCh38-2020-A and Visium Human Transcriptome Probe Set version 1.0 GRCh38-2020-A.csv annotations. Loupe files (generated from raw data using 10X Genomics Cloud Analysis software) were analysed using loupe browser and count matrices were imported into R for analysis with *Seurat*. Clustering and spatial enhancement was performed using *BayesSpace* according to author’s instruction. To define tumour-infiltrating immune cells within the spatial framework, gene expression signature lists were used from the R package *ImSig* ^2^ for immune cells (B cells, T cells, NK cells, neutrophils, macrophages and monocytes). ImSig algorithm assigns spatial spot to a particular subset of immune cells based on the expression of 20-25 most significant genes for this type of cells. Such relatively unbiased method allowed to more precisely locate tumour-infilitrating immune cells. Loupe browser version 6.0.0 (10x Genomics) was used for analysis of spatial data. ConsensusPath-DB online software was used to functionally annotate the set of significant (p<0.05) DEGs.

**Methods of SENTIS+ Cancer discovery panel (BGI Genomics).**

The purity and concentration of DNA obtained from mesothelioma tissues were evaluated using the Qubit 2.0 Fluorometer with Quant-IT dsDNA HS Assay Kit (Thermo Fisher Scientific), respectively. The quality of genomic DNA was assessed through agarose gel electrophoresis. To construct DNA libraries, 400ng of DNA was sheared by Covatris to produce fragments with a peak of 200-300 bps, followed by end repair, A-tailing, and ligation to the MGI-indexed adapters according to the standard library construction protocol. Target enrichment was carried out on 10000 exons and 9383 introns of 688 cancer-related genes in solid tumor, representing approximately 2.37 Mb of the human genome. Sequencing was conducted using 2 × 100 bp paired-end reads on the DNBSEQ-G400 platform (DNA nanoball sequencing platform by MGI-Tech). The primary raw reads underwent initial processing by removing adaptors and filtering low-quality reads using SOAPnuke (http://soap.genomics.org.cn/). Clean reads were then aligned to the human reference GRCh37 using the BWA aligner (v0.6.2-r126). PCR duplications were eliminated using PICARD (v1.98). Local realignment and base quality score recalibrations were performed using GATK (v2.3-9), and poorly mapped reads were subsequently removed. SNVs were identified using Mutect and SOMATK-SNV (developed by BGI), while InDels were detected using GATK and SOMATK-INDEL (developed by BGI). The CNV analysis was conducted using CONTRA (v2.0.4) based on off-target sequencing data, which was utilized as low-depth whole-genome sequencing data. Oviz-Bio and Convaq packages were used for data visualisation.

***Figure S1****.* H&E-stained tissues mounted onto Visium spatial slide. This image stresses the differences in size of the resected tissue as well as differences in the amount of adjacent adipose tissues due to variability in biopsy techniques.

***Figure S2****.* Kaplan-Meier curves depicting the differences in overall survival in patients with malignant pleural mesothelioma based on the expression of HAVCR2 gene and number of tumour-infiltrating macrophages. The plot was generated using TIMER 2.0 ^10^ online software.

***Figure S3****.* Violin plots depicting the association of mutated PTCH1 gene on infiltration of CD8+ T cells, CD4+ T cells, NK cells, macrophages, B cells. The plots were generated using TIMER 2.0 ^10^ online software. WT – wild type.

**Table S1.** Clinical characteristics of the recruited patients**.** ECOG PS – Eastern Cooperation Oncology Group Performance Status; C1D1 – cycle 1: day 1; ICI – immune-checkpoint inhibitors; CR – complete response; PR – partial response; DP – disease progression; SD – stable disease; G – grade; N/A – not applicable

| ID | A1 | B1 | C1 | D1 |
| --- | --- | --- | --- | --- |
| Age | 72 | 67 | 77 | 78 |
| Ethnicity | Caucasian | Caucasian | Caucasian | Caucasian |
| Gender | Male | Male | Male | Male |
| Pre-treatment ECOG PS | 0 | 0 | 1 | 1 |
| Histology | Left epithelioid mesothelioma | Right epithelioid mesothelioma | Left epithelioid mesothelioma | Right sarcomatoid mesothelioma |
| Concurrent medications | Escitalopram, simvastatin, metoprolol, aspirin, insulin isophane, isosorbide mononitrate, metformin | Escitalopram | Coloxyl senna, Endone, paracetamol | Pregabalin, Endone, Paracetamol, Meloxicam |
| Pre-treatment BMI | 23.24 | 30.25 | 21.73 | 20.42 |
| Smoking status | Non-smoker | Non-smoker | Non-smoker | Ex-smoker (40 pack/years) |
| Treatment prior to ICI | - Aug 2020 – Oct 2020: Carboplatin + Pemetrexed + Bevacizumab (4 cycles). - Oct 2020 – Apr 2021: Bevacizumab (maintenance). | - Jun 2019 – Aug: Cisplatin + Pemetrexed (3 cycles). - April 2019 – mesothelioma resection followed by radiation therapy. | - Feb 2019 – April 2019: Cisplatin + Pemetrexed + Bevacizumab (44 cycles). - June 2019 – June 2021: Bevacizumab | - Sep 2020 – Oct 2020 – Radiation therapy for R lung (50 Gy in 20 fractions). |
| Efficacy outcomes | CR | DP | SD | DP |
| irAEs | Pneumonitis G3-4 (Nov-21), Pneumonitis flare-up (Feb-22) | None | Hepatitis G4 | Pneumonitis G3-4 |
| Treatment of irAEs | Prednisone (Nov-21), Prednisone + mycophenolate (Feb-22) | N/A | Prednisone | Prednisone |

**Table S2.** GO terms enriched by the significantly upregulated gene in non-responders.

| **GO Term** | **Term name** | **List of upregulated genes** | **Q-value** |
| --- | --- | --- | --- |
| GO:0030198 | extracellular matrix organization | LUM; TIMP2; CCN2; BGN; AEBP1; FBLN1; SERPINE1; FKBP10; THBS1; SPARC; COL1A2; ITGB1; ITGB5; POSTN; COL5A1; CAPG; SFRP2; FN1; SPP1; COL1A1; COL6A1; TGFBI; COL6A2 | 1.42E-19 |
| GO:0070062 | extracellular exosome | ACTA2; HLA-A; IGHA1; FCGR3A; IGHG1; BGN; HSPA1A; KRT7; S100A11; FBLN1; THBS1; LUM; TXN; CAPG; SERPINE1; SPP1; ITGB1; SPON2; ITGB5; CTSB; HLA-DRB1; HLA-DRB5; IGFBP2; TGFBI; LGALS1; HBA2; JCHAIN; SFRP1; MYL12B; FN1; FSTL1; CD14; COL1A2; PRSS23; COL6A1; AEBP1; COL6A2 | 3.35E-16 |
| GO:0005788 | endoplasmic reticulum lumen | FKBP10; FSTL1; FN1; PLAUR; PRSS23; CALU; THBS1; IGFBP3; COL5A1; COL1A2; COL1A1; COL6A1; SPP1; LGALS1; COL6A2 | 5.21E-12 |
| GO:0005178 | integrin binding | SFRP2; ITGB1; ITGB5; CCN2; FN1; THBS1; COL5A1; SPP1; TGFBI; TIMP2 | 2.39E-09 |
| GO:0048584 | positive regulation of response to stimulus | TIMP3; TIMP2; CCN2; PLAUR; HLA-A; IGHA1; FCGR3A; IGHG1; HSPA1A; SERPINE1; TXN; THBS1; VSIG4; ITGB1; CTSB; HLA-DRB1; HLA-DRB5; IGFBP3; LGALS1; SFRP2; SFRP1; SFRP4; FN1; CCL18; C1QC; C1QB; C1QA; CD14; COL1A1; HLA-DQA1 | 5.71E-08 |
| GO:0030199 | collagen fibril organization | SFRP2; FKBP10; COL5A1; AEBP1; COL1A1; COL1A2; LUM | 9.20E-08 |
| GO:0001944 | vasculature development | ACTA2; SFRP2; SFRP1; FN1; CCN2; THBS2; THBS1; ITGB1; SPARC; COL5A1; COL1A2; COL1A1; TGFBI; FKBP10; CALD1; SERPINE1 | 1.66E-07 |
| GO:0072358 | cardiovascular system development | ACTA2; SFRP2; SFRP1; FN1; CCN2; THBS2; THBS1; ITGB1; SPARC; COL5A1; COL1A2; COL1A1; TGFBI; FKBP10; CALD1; SERPINE1 | 1.66E-07 |
| GO:0016477 | cell migration | CCN2; IGHA1; S100A11; FSTL1; SERPINE1; NBL1; THBS1; SPARC; ITGB1; ITGB5; DPYSL3; POSTN; COL5A1; JCHAIN; SFRP2; SFRP1; FN1; IGFBP3; CCL18; COL1A2; COL1A1; CTHRC1 | 4.73E-07 |
| GO:0034097 | response to cytokine | ITGB1; SFRP1; TIMP3; DPYSL3; FN1; CCL18; HLA-A; HLA-DRB1; IGHG1; HSPA1A; HLA-DRB5; SPARC; POSTN; CD14; COL1A2; COL1A1; THBS1; HLA-DQA1; TIMP2 | 8.54E-07 |
| GO:0071310 | cellular response to organic substance | CCN2; HLA-A; IGHG1; HSPA1A; FSTL1; SERPINE1; NBL1; THBS1; SPP1; ITGB1; ITGB5; DPYSL3; CTSB; HLA-DRB1; HLA-DRB5; IGFBP2; POSTN; LGALS1; SFRP2; SFRP1; SFRP4; FN1; CCL18; CD14; COL1A2; COL1A1; COL6A1; HLA-DQA1 | 1.16E-06 |
| GO:0048407 | platelet-derived growth factor binding | COL6A1; COL5A1; COL1A2; COL1A1 | 7.12E-07 |
| GO:0045595 | regulation of cell differentiation | TIMP2; CCN2; HSPA1A; FBLN1; SERPINE1; MYL9; NBL1; THBS1; DPYSL3; HLA-DRB1; POSTN; COL5A1; TMEM176B; LGALS1; SFRP2; SFRP1; FN1; SFRP4; IGFBP3; C1QC; SPP1; COL1A1; CTHRC1 | 2.08E-06 |
| GO:0010812 | negative regulation of cell-substrate adhesion | THBS1; POSTN; COL1A1; FBLN1; LGALS1; SERPINE1 | 2.15E-05 |
| GO:2000026 | regulation of multicellular organismal development | TIMP2; CCN2; HSPA1A; SERPINE1; MYL9; AP2S1; NBL1; THBS2; THBS1; SPARC; ITGB1; DPYSL3; HLA-DRB1; COL5A1; TMEM176B; LGALS1; SFRP2; SFRP1; FN1; SFRP4; C1QC; SPP1; COL1A1; CTHRC1 | 2.97E-06 |
| GO:0031410 | cytoplasmic vesicle | TIMP3; TIMP2; PLAUR; HLA-A; BGN; HSPA1A; S100A11; SERPINE1; AP2S1; CAPG; THBS2; THBS1; SPARC; ITGB1; ITGB5; DPYSL3; CTSB; HLA-DRB1; HLA-DRB5; IGFBP2; HBA2; FN1; CALU; CD14; COL1A1; HLA-DQA1 | 1.80E-06 |
| GO:0072359 | circulatory system development | ACTA2; SFRP2; SFRP1; FN1; CCN2; ITGB1; THBS2; THBS1; FHL2; SPARC; COL5A1; COL1A2; COL1A1; TGFBI; FKBP10; CALD1; SERPINE1 | 3.47E-06 |
| GO:0042060 | wound healing | ITGB1; FKBP10; FN1; PLAUR; THBS1; POSTN; SPARC; COL5A1; COL1A2; COL1A1; FBLN1; SERPINE1; MYL9 | 3.47E-06 |
| GO:0048585 | negative regulation of response to stimulus | SFRP2; IGFBP2; SFRP1; TIMP3; TIMP2; SFRP4; TXN; NBL1; HLA-A; HLA-DRB1; CTHRC1; HSPA1A; THBS1; FHL2; IGFBP3; CD14; SPP1; VSIG4; PLAUR; FBLN1; SERPINE1 | 3.69E-06 |
| GO:0070848 | response to growth factor | SFRP2; ITGB1; SFRP1; ITGB5; SFRP4; CCN2; NBL1; THBS1; POSTN; HSPA1A; COL1A2; COL1A1; FSTL1; LUM | 4.07E-06 |
| GO:0002684 | positive regulation of immune system process | IGHA1; VSIG4; SERPINE1; C1QC; HLA-A; HLA-DRB1; FCGR3A; C1QB; C1QA; HLA-DRB5; IGFBP2; CD14; CTSB; IGHG1; LGALS1; THBS1; HLA-DQA1; HSPA1A | 5.84E-06 |
| GO:0048523 | negative regulation of cellular process | TIMP3; TIMP2; CCN2; PLAUR; VSIG4; HSPA1A; AEBP1; APOC1; S100A11; FBLN1; FSTL1; SERPINE1; TXN; NBL1; THBS1; FHL2; SPARC; ITGB1; DPYSL3; HLA-DRB1; IGFBP2; IGFBP3; COL5A1; TMEM176B; LGALS1; CAPG; SFRP2; SFRP1; SFRP4; POSTN; MAGED1; C1QC; CD14; SPP1; COL1A1; TGFBI; CTHRC1 | 9.18E-06 |
| GO:0002020 | protease binding | ITGB1; TIMP3; TIMP2; FN1; COL1A2; COL1A1; SERPINE1 | 4.48E-06 |
| GO:0007178 | transmembrane receptor protein serine/threonine kinase signaling pathway | ITGB1; SFRP2; SFRP1; ITGB5; SFRP4; NBL1; THBS1; HSPA1A; COL1A2; FSTL1 | 4.47E-05 |
| GO:0050776 | regulation of immune response | ITGB1; IGHA1; VSIG4; FCGR3A; HLA-A; HLA-DRB1; C1QC; C1QB; C1QA; HLA-DRB5; IGHG1; CD14; COL1A2; COL1A1; CTSB; HLA-DQA1; HSPA1A | 1.06E-05 |
| GO:0007162 | negative regulation of cell adhesion | VSIG4; HLA-DRB1; THBS1; POSTN; COL1A1; TGFBI; FBLN1; LGALS1; SERPINE1 | 1.19E-05 |
| GO:0051241 | negative regulation of multicellular organismal process | SFRP2; SFRP1; DPYSL3; FN1; THBS2; PLAUR; VSIG4; HLA-DRB1; C1QC; THBS1; SPARC; COL5A1; SPP1; APOC1; LGALS1; TMEM176B; SERPINE1 | 1.19E-05 |
| GO:0008285 | negative regulation of cell proliferation | SFRP2; SFRP1; TIMP2; SFRP4; MAGED1; VSIG4; HLA-DRB1; SPARC; THBS1; IGFBP3; HSPA1A; S100A11; FBLN1 | 4.47E-05 |
| GO:0007167 | enzyme linked receptor protein signaling pathway | PLAUR; SFRP2; AP2S1; SFRP1; ITGB5; SFRP4; CCN2; NBL1; THBS1; IGFBP2; IGFBP3; HSPA1A; ITGB1; COL1A2; COL1A1; FSTL1 | 1.22E-05 |
| GO:0071363 | cellular response to growth factor stimulus | ITGB1; SFRP2; SFRP1; ITGB5; SFRP4; CCN2; NBL1; THBS1; POSTN; HSPA1A; COL1A2; COL1A1; FSTL1 | 4.47E-05 |
| GO:0001649 | osteoblast differentiation | SFRP2; SFRP1; FHL2; IGFBP3; SPP1; COL1A1; COL6A1; CTHRC1 | 1.45E-05 |
| GO:0010810 | regulation of cell-substrate adhesion | SFRP1; FN1; THBS1; POSTN; COL1A1; FBLN1; LGALS1; SERPINE1 | 1.49E-05 |
| GO:0048514 | blood vessel morphogenesis | SFRP2; ITGB1; SFRP1; CCN2; FN1; THBS2; THBS1; SPARC; TGFBI; FKBP10; CALD1; SERPINE1 | 1.71E-05 |
| GO:0045597 | positive regulation of cell differentiation | SFRP2; SFRP1; SFRP4; DPYSL3; FN1; NBL1; CCN2; HLA-DRB1; CTHRC1; IGFBP3; HSPA1A; COL1A1; LGALS1; SERPINE1; TIMP2 | 7.24E-05 |
| GO:0007160 | cell-matrix adhesion | ITGB1; SFRP1; ITGB5; CCN2; FN1; THBS1; POSTN; SERPINE1 | 2.28E-05 |
| GO:0051240 | positive regulation of multicellular organismal process | SFRP2; ITGB1; CCN2; DPYSL3; FN1; NBL1; HLA-A; HLA-DRB1; CTHRC1; HSPA1A; SERPINE1; SPARC; POSTN; CD14; SPP1; COL1A1; LGALS1; THBS1; LUM; TIMP2 | 2.32E-05 |
| GO:0042611 | MHC protein complex | HLA-A; HLA-DRB1; HLA-DQA1; HLA-DRB5 | 2.78E-05 |
| GO:0051336 | regulation of hydrolase activity | SFRP2; ITGB1; SFRP1; TIMP3; CCN2; FN1; CCL18; PLAUR; TPM2; SERPINE1; IGFBP3; HSPA1A; APOC1; FBLN1; THBS1; CNN3; TIMP2 | 3.12E-05 |
| GO:0061448 | connective tissue development | ACTA2; SFRP2; CCN2; BGN; COL5A1; COL1A1; TGFBI; LUM | 3.25E-05 |
| GO:0071560 | cellular response to transforming growth factor beta stimulus | ITGB1; SFRP1; ITGB5; THBS1; POSTN; HSPA1A; COL1A2; COL1A1 | 3.25E-05 |
| GO:0007369 | gastrulation | SFRP2; ITGB1; SFRP1; ITGB5; FN1; COL5A1; COL6A1 | 3.72E-05 |
| GO:0080134 | regulation of response to stress | SFRP2; TXN; SFRP1; CCN2; PLAUR; HLA-A; HLA-DRB1; C1QC; C1QB; C1QA; IGHG1; CTSB; CD14; ITGB1; SPP1; VSIG4; THBS1; SERPINE1; HSPA1A | 3.98E-05 |
| GO:0005783 | endoplasmic reticulum | THBS1; FSTL1; HLA-DRB1; PRSS23; FN1; PLAUR; HLA-A; APOC1; CALU; HLA-DRB5; IGFBP3; HSPA1A; COL5A1; COL1A2; FKBP10; COL1A1; COL6A1; SPP1; LGALS1; COL6A2; HLA-DQA1 | 3.44E-05 |
| GO:0071556 | integral component of lumenal side of endoplasmic reticulum membrane | HLA-A; HLA-DRB1; HLA-DQA1; HLA-DRB5 | 8.42E-05 |
| GO:0010941 | regulation of cell death | SFRP2; TXN; SFRP1; TIMP3; SFRP4; CCN2; PLAUR; CTSB; C1QA; THBS1; FHL2; IGFBP3; HSPA1A; ITGB1; MAGED1; FSTL1; LGALS1; HBA2; SERPINE1 | 4.25E-05 |
| GO:0051094 | positive regulation of developmental process | SFRP2; ITGB1; SFRP1; SFRP4; DPYSL3; FN1; NBL1; CCN2; HLA-DRB1; CTHRC1; THBS1; IGFBP3; HSPA1A; COL1A1; LGALS1; SERPINE1; TIMP2 | 4.54E-05 |
| GO:2000145 | regulation of cell motility | SFRP2; ITGB1; SFRP1; DPYSL3; FN1; POSTN; NBL1; THBS1; IGFBP3; SPARC; COL1A1; S100A11; FBLN1; SERPINE1 | 4.97E-05 |
| GO:0050778 | positive regulation of immune response | IGHA1; VSIG4; C1QC; CTSB; HLA-DRB1; FCGR3A; C1QB; C1QA; HLA-DQA1; CD14; IGHG1; HLA-DRB5; HLA-A; HSPA1A | 0.000201 |
| GO:0051093 | negative regulation of developmental process | SFRP2; SFRP1; DPYSL3; THBS2; C1QC; SERPINE1; POSTN; SPARC; COL5A1; SPP1; TMEM176B; FBLN1; THBS1; LGALS1 | 5.65E-05 |
| GO:0071345 | cellular response to cytokine stimulus | ITGB1; SFRP1; DPYSL3; FN1; CCL18; HLA-A; HLA-DRB1; IGHG1; HLA-DRB5; POSTN; HSPA1A; COL1A2; COL1A1; THBS1; HLA-DQA1 | 0.000201 |
| GO:0009968 | negative regulation of signal transduction | SFRP2; IGFBP2; SFRP1; TIMP3; TIMP2; SFRP4; NBL1; CTHRC1; HSPA1A; THBS1; FHL2; IGFBP3; CD14; PLAUR; FBLN1; SERPINE1 | 0.000231 |
| GO:0098883 | synapse pruning | C1QC; C1QB; C1QA | 7.43E-05 |
| GO:0048522 | positive regulation of cellular process | ACTA2; TIMP3; TIMP2; CCN2; PLAUR; HLA-A; IGHA1; IGHG1; HSPA1A; APOC1; S100A11; FBLN1; THBS1; LUM; TXN; CAPG; NBL1; SERPINE1; SPARC; ITGB1; DPYSL3; HLA-DRB1; IGFBP2; IGFBP3; LGALS1; HBA2; SFRP2; SFRP1; SFRP4; FN1; POSTN; CCL18; C1QA; CD14; SPP1; COL1A1; CTHRC1 | 8.86E-05 |
| GO:0042127 | regulation of cell proliferation | SFRP2; SFRP1; SFRP4; CCN2; FN1; MAGED1; HLA-A; HLA-DRB1; SPARC; THBS1; IGFBP2; IGFBP3; HSPA1A; CTHRC1; S100A11; FBLN1; VSIG4; TIMP2 | 9.19E-05 |
| GO:0006897 | endocytosis | ITGB1; AP2S1; SFRP4; IGHA1; FCGR3A; IGHG1; THBS1; SPARC; CD14; APOC1; HBA2; SERPINE1; JCHAIN | 0.00025 |
| GO:0010647 | positive regulation of cell communication | ITGB1; SFRP2; SFRP1; SFRP4; CCN2; FN1; TXN; CCL18; PLAUR; HLA-DRB1; CD14; THBS1; IGFBP3; HSPA1A; SPP1; COL1A1; TIMP3; LGALS1; TIMP2 | 0.00025 |
| GO:0009967 | positive regulation of signal transduction | ITGB1; SFRP2; SFRP1; SFRP4; CCN2; FN1; TXN; CCL18; PLAUR; HLA-DRB1; CD14; THBS1; IGFBP3; HSPA1A; COL1A1; TIMP3; LGALS1; TIMP2 | 0.00025 |
| GO:0023056 | positive regulation of signaling | SFRP2; TXN; SFRP1; SFRP4; CCN2; FN1; CCL18; PLAUR; HLA-DRB1; CD14; THBS1; IGFBP3; HSPA1A; ITGB1; SPP1; COL1A1; TIMP3; LGALS1; TIMP2 | 0.000106 |
| GO:0008625 | extrinsic apoptotic signaling pathway via death domain receptors | SFRP2; SFRP1; TIMP3; SERPINE1; THBS1 | 0.00025 |
| GO:0009966 | regulation of signal transduction | TIMP3; TIMP2; CCN2; PLAUR; HSPA1A; FBLN1; FSTL1; SERPINE1; TXN; NBL1; THBS1; FHL2; ITGB1; HLA-DRB1; IGFBP2; IGFBP3; LGALS1; SFRP2; SFRP1; FN1; SFRP4; POSTN; CCL18; CD14; COL1A1; CTHRC1 | 0.000106 |
| GO:0099503 | secretory vesicle | TIMP3; DPYSL3; FN1; PLAUR; CTSB; SPARC; THBS2; CD14; THBS1; HSPA1A; COL1A1; S100A11; SERPINE1; TIMP2 | 0.000118 |
| GO:0051270 | regulation of cellular component movement | SFRP2; ITGB1; SFRP1; DPYSL3; FN1; POSTN; NBL1; THBS1; IGFBP3; SPARC; COL1A1; S100A11; FBLN1; SERPINE1 | 0.000109 |
| GO:0090177 | establishment of planar polarity involved in neural tube closure | SFRP2; SFRP1; CTHRC1 | 0.000109 |
| GO:0030669 | clathrin-coated endocytic vesicle membrane | AP2S1; HLA-DRB1; HLA-DQA1; HLA-DRB5 | 9.42E-05 |
| GO:0005584 | collagen type I trimer | COL1A2; COL1A1 | 9.42E-05 |
| GO:0051271 | negative regulation of cellular component movement | SFRP2; SFRP1; DPYSL3; NBL1; THBS1; IGFBP3; FBLN1; SERPINE1 | 0.000289 |
| GO:0030139 | endocytic vesicle | AP2S1; ITGB5; HLA-A; HLA-DRB1; HLA-DRB5; SPARC; HBA2; HLA-DQA1 | 0.000118 |
| GO:0033273 | response to vitamin | POSTN; SPARC; SFRP1; SPP1; COL1A1 | 0.000138 |
| GO:0010648 | negative regulation of cell communication | SFRP2; IGFBP2; SFRP1; TIMP3; TIMP2; SFRP4; NBL1; CTHRC1; HSPA1A; THBS1; FHL2; IGFBP3; CD14; PLAUR; FBLN1; SERPINE1 | 0.000336 |
| GO:0051017 | actin filament bundle assembly | ITGB1; SFRP1; ITGB5; DPYSL3; CCN2; CALD1 | 0.000149 |
| GO:0023057 | negative regulation of signaling | SFRP2; IGFBP2; SFRP1; TIMP3; TIMP2; SFRP4; NBL1; CTHRC1; HSPA1A; THBS1; FHL2; IGFBP3; CD14; PLAUR; FBLN1; SERPINE1 | 0.000149 |
| GO:0035987 | endodermal cell differentiation | COL6A1; COL5A1; ITGB5; FN1 | 0.000149 |
| GO:0042249 | establishment of planar polarity of embryonic epithelium | SFRP2; SFRP1; CTHRC1 | 0.000337 |
| GO:0040013 | negative regulation of locomotion | SFRP2; SFRP1; DPYSL3; NBL1; THBS1; IGFBP3; FBLN1; SERPINE1 | 0.000155 |
| GO:0022603 | regulation of anatomical structure morphogenesis | SFRP2; ITGB1; AP2S1; SFRP1; FN1; THBS2; THBS1; POSTN; SPARC; COL5A1; SPP1; CTHRC1; FBLN1; SERPINE1 | 0.000155 |
| GO:0061572 | actin filament bundle organization | ITGB1; SFRP1; ITGB5; DPYSL3; CCN2; CALD1 | 0.000337 |
| GO:0042613 | MHC class II protein complex | HLA-DRB1; HLA-DQA1; HLA-DRB5 | 0.00015 |
| GO:0090092 | regulation of transmembrane receptor protein serine/threonine kinase signaling pathway | SFRP2; SFRP1; SFRP4; NBL1; THBS1; HSPA1A; FSTL1 | 0.00043 |
| GO:0006508 | proteolysis | SFRP2; SFRP1; TIMP3; CCN2; FN1; PLAUR; VSIG4; C1QC; C1QB; C1QA; IGHG1; CTSB; HSPA1A; AEBP1; PRSS23; FBLN1; THBS1; SERPINE1; TIMP2 | 0.000208 |
| GO:0010646 | regulation of cell communication | TIMP3; TIMP2; CCN2; PLAUR; HSPA1A; FBLN1; FSTL1; SERPINE1; TXN; NBL1; THBS1; FHL2; ITGB1; HLA-DRB1; IGFBP2; IGFBP3; LGALS1; SFRP2; SFRP1; FN1; SFRP4; POSTN; CCL18; CD14; SPP1; COL1A1; CTHRC1 | 0.000208 |
| GO:0060205 | cytoplasmic vesicle lumen | TIMP3; TIMP2; FN1; THBS1; SPARC; S100A11; HBA2; SERPINE1 | 0.000187 |
| GO:0016485 | protein processing | VSIG4; C1QC; C1QB; C1QA; IGHG1; AEBP1; THBS1; SERPINE1 | 0.000456 |
| GO:0010942 | positive regulation of cell death | ITGB1; SFRP2; SFRP1; TIMP3; SFRP4; CCN2; PLAUR; C1QA; THBS1; IGFBP3; HBA2 | 0.000456 |
| GO:0007599 | hemostasis | FN1; PLAUR; THBS1; COL1A2; COL1A1; FBLN1; SERPINE1; MYL9 | 0.000247 |
| GO:0002455 | humoral immune response mediated by circulating immunoglobulin | HLA-DRB1; IGHA1; C1QC; C1QB; C1QA; IGHG1 | 0.000269 |
| GO:0001706 | endoderm formation | COL6A1; COL5A1; ITGB5; FN1 | 0.000272 |
| GO:0006954 | inflammatory response | FN1; CCL18; VSIG4; HLA-DRB1; C1QC; C1QB; C1QA; IGHG1; CD14; SPP1; THBS1; SERPINE1 | 0.000276 |
| GO:0002478 | antigen processing and presentation of exogenous peptide antigen | AP2S1; ITGB5; HLA-A; HLA-DRB1; HLA-DRB5; HLA-DQA1 | 0.000276 |
| GO:0002683 | negative regulation of immune system process | SFRP1; NBL1; HLA-A; HLA-DRB1; C1QC; THBS1; CD14; TMEM176B; VSIG4 | 0.000276 |
| GO:0007229 | integrin-mediated signaling pathway | ITGB1; FBLN1; ITGB5; FN1; CCN2 | 0.000292 |
| GO:0016192 | vesicle-mediated transport | ITGB1; THBS1; AP2S1; TIMP3; SFRP4; FN1; TIMP2; PLAUR; CTSB; IGHA1; FCGR3A; HSPA1A; IGHG1; SPARC; CD14; APOC1; S100A11; HBA2; SERPINE1; JCHAIN | 0.000308 |
| GO:0051216 | cartilage development | SFRP2; CCN2; BGN; COL1A1; TGFBI; LUM | 0.000313 |
| GO:0090287 | regulation of cellular response to growth factor stimulus | SFRP2; SFRP1; SFRP4; NBL1; THBS1; HSPA1A; FSTL1 | 0.000334 |
| GO:0051128 | regulation of cellular component organization | CCN2; PLAUR; HSPA1A; AEBP1; APOC1; FBLN1; SERPINE1; AP2S1; THBS1; SPARC; DPYSL3; POSTN; COL5A1; LGALS1; CAPG; SFRP2; SFRP1; FN1; SFRP4; IGFBP3; CD14; SPP1 | 0.000334 |
| GO:0001501 | skeletal system development | SFRP2; SFRP1; SFRP4; CCN2; BGN; COL1A2; COL1A1; TGFBI; LUM | 0.000339 |
| GO:0098657 | import into cell | ITGB1; AP2S1; SFRP4; IGHA1; FCGR3A; IGHG1; THBS1; SPARC; CD14; APOC1; HBA2; SERPINE1; JCHAIN | 0.000352 |
| GO:0034284 | response to monosaccharide | CCN2; HLA-DRB1; THBS1; SPARC; LGALS1; COL6A2 | 0.000872 |
| GO:0030449 | regulation of complement activation | VSIG4; C1QC; C1QB; C1QA; IGHG1 | 0.000872 |
| GO:0043067 | regulation of programmed cell death | ITGB1; SFRP2; SFRP1; TIMP3; SFRP4; CCN2; PLAUR; CTSB; THBS1; FHL2; IGFBP3; HSPA1A; MAGED1; LGALS1; FSTL1; SERPINE1 | 0.000872 |
| GO:0071748 | monomeric IgA immunoglobulin complex | IGHA1; JCHAIN | 0.000401 |
| GO:0007161 | calcium-independent cell-matrix adhesion | ITGB1; FN1 | 0.000872 |
| GO:2000257 | regulation of protein activation cascade | VSIG4; C1QC; C1QB; C1QA; IGHG1 | 0.000444 |
| GO:0012507 | ER to Golgi transport vesicle membrane | HLA-A; HLA-DRB1; HLA-DQA1; HLA-DRB5 | 0.000401 |
| GO:1902531 | regulation of intracellular signal transduction | ITGB1; SFRP2; SFRP1; TIMP3; CCN2; FN1; TXN; CCL18; PLAUR; HLA-DRB1; HSPA1A; THBS1; FHL2; IGFBP3; CD14; FBLN1; LGALS1; TIMP2 | 0.000895 |
| GO:0032355 | response to estradiol | IGFBP2; POSTN; SFRP1; CCN2; COL1A1 | 0.000491 |
| GO:0050865 | regulation of cell activation | IGHA1; CCN2; HLA-A; HLA-DRB1; IGHG1; THBS1; IGFBP2; SFRP1; LGALS1; VSIG4 | 0.000492 |
| GO:0045765 | regulation of angiogenesis | ITGB1; SFRP2; SFRP1; THBS2; THBS1; SPARC; SERPINE1 | 0.000965 |
| GO:0045055 | regulated exocytosis | TIMP3; TIMP2; FN1; PLAUR; CTSB; SPARC; HSPA1A; THBS1; CD14; S100A11; SERPINE1 | 0.000555 |
| GO:0051604 | protein maturation | VSIG4; C1QC; C1QB; C1QA; IGHG1; AEBP1; THBS1; SERPINE1 | 0.000619 |
| GO:0044344 | cellular response to fibroblast growth factor stimulus | POSTN; SFRP1; THBS1; CCN2; COL1A1 | 0.000619 |
| GO:0097191 | extrinsic apoptotic signaling pathway | SFRP2; SFRP1; TIMP3; THBS1; HSPA1A; SERPINE1 | 0.000657 |
| GO:0071746 | IgA immunoglobulin complex, circulating | IGHA1; JCHAIN | 0.000546 |
| GO:0071749 | polymeric IgA immunoglobulin complex | IGHA1; JCHAIN | 0.000546 |
| GO:0034612 | response to tumor necrosis factor | SFRP1; CCL18; HSPA1A; THBS1; POSTN; CD14; COL1A1 | 0.001283 |
| GO:0045785 | positive regulation of cell adhesion | SFRP2; SFRP1; FN1; PLAUR; HLA-A; HLA-DRB1; IGFBP2; LGALS1 | 0.000669 |
| GO:0050867 | positive regulation of cell activation | IGHA1; CCN2; HLA-A; HLA-DRB1; IGHG1; THBS1; IGFBP2; LGALS1 | 0.001283 |
| GO:0009743 | response to carbohydrate | CCN2; HLA-DRB1; THBS1; SPARC; LGALS1; COL6A2 | 0.000672 |
| GO:0005520 | insulin-like growth factor binding | IGFBP2; IGFBP3; CCN2 | 0.001095 |
| GO:0002526 | acute inflammatory response | FN1; VSIG4; C1QC; IGHG1; C1QA; C1QB | 0.001437 |
| GO:0007015 | actin filament organization | ITGB1; SFRP1; ITGB5; DPYSL3; CCN2; TPM2; CAPG; CALD1 | 0.000788 |
| GO:0034774 | secretory granule lumen | TIMP3; TIMP2; FN1; THBS1; SPARC; S100A11; SERPINE1 | 0.000782 |
| GO:0007492 | endoderm development | COL6A1; COL5A1; ITGB5; FN1 | 0.000799 |
| GO:0002920 | regulation of humoral immune response | VSIG4; C1QC; C1QB; C1QA; IGHG1 | 0.001534 |
| GO:0014812 | muscle cell migration | S100A11; IGFBP3; SERPINE1; POSTN | 0.000867 |
| GO:0046903 | secretion | SFRP1; TIMP3; CCN2; FN1; PLAUR; CTSB; HLA-DRB1; CD14; THBS1; SPARC; HSPA1A; SPP1; S100A11; SERPINE1; TIMP2 | 0.000867 |
| GO:1901342 | regulation of vasculature development | ITGB1; SFRP2; SFRP1; THBS2; THBS1; SPARC; SERPINE1 | 0.001605 |
| GO:0051346 | negative regulation of hydrolase activity | SFRP2; TIMP3; TIMP2; CNN3; PLAUR; THBS1; APOC1; SERPINE1 | 0.001606 |
| GO:0048545 | response to steroid hormone | SFRP1; CCN2; THBS1; IGFBP2; SPARC; SPP1; COL1A1 | 0.000903 |
| GO:0001817 | regulation of cytokine production | FN1; HLA-A; HLA-DRB1; CD14; SERPINE1; POSTN; HSPA1A; VSIG4; THBS1; LUM | 0.00095 |
| GO:0045596 | negative regulation of cell differentiation | SFRP2; SFRP1; DPYSL3; C1QC; POSTN; COL5A1; SPP1; TMEM176B; FBLN1; LGALS1 | 0.001683 |
| GO:0005509 | calcium ion binding | MYL12B; FKBP10; THBS2; CALU; THBS1; SPARC; S100A11; FBLN1; FSTL1; MYL9 | 0.001751 |
| GO:0006936 | muscle contraction | ACTA2; ITGB5; MYL12B; CCN2; TPM2; CALD1; MYL9 | 0.001054 |
| GO:0044409 | entry into host | ITGB1; HSPA1A; HLA-DRB1; ITGB5; LGALS1 | 0.001788 |
| GO:0051806 | entry into cell of other organism involved in symbiotic interaction | ITGB1; HSPA1A; HLA-DRB1; ITGB5; LGALS1 | 0.001788 |
| GO:0051828 | entry into other organism involved in symbiotic interaction | ITGB1; HSPA1A; HLA-DRB1; ITGB5; LGALS1 | 0.001089 |
| GO:0019724 | B cell mediated immunity | HLA-DRB1; IGHA1; C1QC; C1QB; C1QA; IGHG1 | 0.001826 |
| GO:0034109 | homotypic cell-cell adhesion | PLAUR; LGALS1; FN1; MYL9 | 0.001174 |
| GO:0006958 | complement activation, classical pathway | IGHA1; C1QC; C1QB; C1QA; IGHG1 | 0.001196 |
| GO:0090288 | negative regulation of cellular response to growth factor stimulus | SFRP2; NBL1; HSPA1A; SFRP1; THBS1 | 0.002016 |
| GO:0002694 | regulation of leukocyte activation | IGHA1; HLA-A; HLA-DRB1; IGHG1; THBS1; IGFBP2; SFRP1; LGALS1; VSIG4 | 0.001269 |
| GO:1903035 | negative regulation of response to wounding | PLAUR; SPP1; SERPINE1; THBS1 | 0.002108 |
| GO:0071773 | cellular response to BMP stimulus | SFRP2; NBL1; SFRP1; SFRP4; FSTL1 | 0.001346 |
| GO:0045637 | regulation of myeloid cell differentiation | SFRP1; HLA-DRB1; C1QC; THBS1; HSPA1A; MYL9 | 0.002176 |
| GO:0060333 | interferon-gamma-mediated signaling pathway | HLA-A; HLA-DRB1; HLA-DQA1; HLA-DRB5 | 0.002178 |
| GO:0031347 | regulation of defense response | VSIG4; CTSB; HLA-DRB1; C1QC; C1QB; C1QA; IGHG1; SERPINE1; CD14; HLA-A; HSPA1A | 0.002192 |
| GO:0006915 | apoptotic process | SFRP2; ITGB1; SFRP1; TIMP3; SFRP4; CCN2; PLAUR; CTSB; HSPA1A; THBS1; FHL2; IGFBP3; CD14; MAGED1; LGALS1; FSTL1; SERPINE1 | 0.001463 |
| GO:1903706 | regulation of hemopoiesis | SFRP1; HLA-DRB1; C1QC; THBS1; HSPA1A; TMEM176B; LGALS1; MYL9 | 0.001477 |
| GO:0031995 | insulin-like growth factor II binding | IGFBP2; IGFBP3 | 0.001751 |
| GO:0071634 | regulation of transforming growth factor beta production | LUM; FN1; THBS1 | 0.002379 |
| GO:0005604 | basement membrane | TGFBI; SPARC; COL5A1; FN1 | 0.001511 |
| GO:0000165 | MAPK cascade | SFRP2; SFRP1; TIMP3; CCN2; FN1; CCL18; HLA-DRB1; THBS1; IGFBP3; FBLN1; TIMP2 | 0.001612 |
| GO:0032101 | regulation of response to external stimulus | NBL1; VSIG4; HLA-DRB1; C1QC; C1QB; C1QA; IGHG1; SPP1; PLAUR; THBS1; SERPINE1 | 0.001684 |
| GO:0002449 | lymphocyte mediated immunity | HLA-DRB1; HLA-A; IGHA1; C1QC; C1QB; C1QA; IGHG1 | 0.001707 |
| GO:2001233 | regulation of apoptotic signaling pathway | SFRP2; SFRP1; TIMP3; PLAUR; THBS1; HSPA1A; SERPINE1 | 0.002705 |
| GO:0019886 | antigen processing and presentation of exogenous peptide antigen via MHC class II | AP2S1; HLA-DRB1; HLA-DQA1; HLA-DRB5 | 0.002705 |
| GO:0060346 | bone trabecula formation | SFRP1; COL1A1 | 0.001837 |
| GO:0060267 | positive regulation of respiratory burst | IGHA1; JCHAIN | 0.002705 |
| GO:0002460 | adaptive immune response based on somatic recombination of immune receptors built from immunoglobulin superfamily domains | HLA-DRB1; HLA-A; IGHA1; C1QC; C1QB; C1QA; IGHG1 | 0.001862 |
| GO:0015629 | actin cytoskeleton | ACTA2; DPYSL3; CNN3; MYL9; TPM2; CAPG; CALD1; MYL12B | 0.001979 |
| GO:1903034 | regulation of response to wounding | ITGB1; PLAUR; SPP1; SERPINE1; THBS1 | 0.002841 |
| GO:0019221 | cytokine-mediated signaling pathway | ITGB1; FN1; CCL18; HLA-A; HLA-DRB1; IGHG1; HLA-DRB5; HSPA1A; COL1A2; HLA-DQA1 | 0.001957 |
| GO:0016504 | peptidase activator activity | SFRP2; FBLN1; FN1 | 0.002707 |
| GO:0043200 | response to amino acid | COL6A1; COL1A2; CCN2; COL1A1 | 0.001957 |
| GO:0030038 | contractile actin filament bundle assembly | ITGB1; SFRP1; ITGB5; CCN2 | 0.00285 |
| GO:0060429 | epithelium development | ACTA2; SFRP2; AP2S1; SFRP1; SFRP4; CNN3; CTSB; FSTL1; COL5A1; KRT7; CTHRC1; SERPINE1; TAGLN | 0.001973 |
| GO:0002495 | antigen processing and presentation of peptide antigen via MHC class II | AP2S1; HLA-DRB1; HLA-DQA1; HLA-DRB5 | 0.001988 |
| GO:0051249 | regulation of lymphocyte activation | IGHA1; HLA-A; HLA-DRB1; IGHG1; IGFBP2; SFRP1; LGALS1; VSIG4 | 0.002929 |
| GO:0002274 | myeloid leukocyte activation | TIMP2; PLAUR; VSIG4; HSPA1A; C1QA; THBS1; CD14; S100A11; CTSB | 0.00207 |
| GO:0031032 | actomyosin structure organization | ITGB1; SFRP1; ITGB5; CCN2; CNN3 | 0.00208 |
| GO:0002424 | T cell mediated immune response to tumor cell | HLA-A; HLA-DRB1 | 0.00208 |
| GO:0051918 | negative regulation of fibrinolysis | SERPINE1; THBS1 | 0.00208 |
| GO:0032395 | MHC class II receptor activity | HLA-DRB1; HLA-DQA1 | 0.002707 |
| GO:0048770 | pigment granule | ITGB1; CTSB; CAPG; CALU | 0.002043 |
| GO:0060548 | negative regulation of cell death | ITGB1; SFRP2; SFRP1; CCN2; TXN; PLAUR; THBS1; FHL2; HSPA1A; FSTL1; SERPINE1 | 0.003262 |
| GO:0002696 | positive regulation of leukocyte activation | IGHA1; HLA-A; HLA-DRB1; IGHG1; THBS1; IGFBP2; LGALS1 | 0.00351 |
| GO:0030195 | negative regulation of blood coagulation | PLAUR; SERPINE1; THBS1 | 0.003607 |
| GO:0043589 | skin morphogenesis | COL1A2; COL1A1 | 0.002511 |
| GO:0042608 | T cell receptor binding | HLA-A; HLA-DRB1 | 0.002829 |
| GO:1900047 | negative regulation of hemostasis | PLAUR; SERPINE1; THBS1 | 0.002632 |
| GO:0071675 | regulation of mononuclear cell migration | NBL1; SERPINE1; THBS1 | 0.003692 |
| GO:0034341 | response to interferon-gamma | CCL18; HLA-A; HLA-DRB1; HLA-DQA1; HLA-DRB5 | 0.003692 |
| GO:2000181 | negative regulation of blood vessel morphogenesis | SPARC; THBS2; SERPINE1; THBS1 | 0.003761 |
| GO:0051272 | positive regulation of cellular component movement | ITGB1; FN1; THBS1; POSTN; SPARC; COL1A1; S100A11; SERPINE1 | 0.003908 |
| GO:0050819 | negative regulation of coagulation | PLAUR; SERPINE1; THBS1 | 0.003918 |
| GO:0061430 | bone trabecula morphogenesis | SFRP1; COL1A1 | 0.002947 |
| GO:0014070 | response to organic cyclic compound | SFRP1; CCN2; THBS1; IGFBP2; POSTN; SPARC; SPP1; COL1A1; LGALS1; LUM | 0.003026 |
| GO:0030665 | clathrin-coated vesicle membrane | AP2S1; HLA-DRB1; HLA-DQA1; HLA-DRB5 | 0.003179 |
| GO:0070371 | ERK1 and ERK2 cascade | TIMP3; CCN2; FN1; CCL18; HLA-DRB1; FBLN1 | 0.004251 |
| GO:0040017 | positive regulation of locomotion | ITGB1; FN1; THBS1; POSTN; SPARC; COL1A1; S100A11; SERPINE1 | 0.003233 |
| GO:0001952 | regulation of cell-matrix adhesion | POSTN; SFRP1; SERPINE1; THBS1 | 0.004369 |
| GO:0032905 | transforming growth factor beta1 production | LUM; THBS1 | 0.00336 |
| GO:0031994 | insulin-like growth factor I binding | IGFBP2; IGFBP3 | 0.003213 |
| GO:0033993 | response to lipid | SFRP1; CCN2; SPARC; THBS1; IGFBP2; POSTN; CD14; SPP1; COL1A1; SERPINE1 | 0.00336 |
| GO:0048705 | skeletal system morphogenesis | SFRP2; SFRP1; SFRP4; CCN2; COL1A1 | 0.00338 |
| GO:0001736 | establishment of planar polarity | SFRP2; AP2S1; SFRP1; CTHRC1 | 0.003502 |
| GO:0001819 | positive regulation of cytokine production | LUM; HLA-A; HSPA1A; THBS1; POSTN; CD14; SERPINE1 | 0.004907 |
| GO:1901343 | negative regulation of vasculature development | SPARC; THBS2; SERPINE1; THBS1 | 0.004907 |
| GO:0051917 | regulation of fibrinolysis | SERPINE1; THBS1 | 0.004956 |
| GO:0032102 | negative regulation of response to external stimulus | NBL1; HLA-DRB1; THBS1; SPP1; PLAUR; SERPINE1 | 0.005123 |
| GO:0030097 | hemopoiesis | ITGB1; SFRP2; SFRP1; HLA-DRB1; C1QC; THBS1; HSPA1A; TMEM176B; LGALS1; MYL9 | 0.005141 |
| GO:0050851 | antigen receptor-mediated signaling pathway | IGHA1; HLA-A; HLA-DRB1; IGHG1; HLA-DRB5; HLA-DQA1 | 0.005196 |
| GO:0045296 | cadherin binding | ITGB1; CNN3; HSPA1A; S100A11; CAPG; CALD1 | 0.004617 |
| GO:0071230 | cellular response to amino acid stimulus | COL6A1; COL1A2; COL1A1 | 0.005672 |
| GO:0060263 | regulation of respiratory burst | IGHA1; JCHAIN | 0.004964 |
| GO:0060026 | convergent extension | SFRP2; SFRP1 | 0.006005 |
| GO:0043292 | contractile fiber | ACTA2; FHL2; CALD1; TPM2; MYL9 | 0.005187 |
| GO:0019731 | antibacterial humoral response | HLA-A; IGHA1; JCHAIN | 0.005022 |
| GO:0051129 | negative regulation of cellular component organization | DPYSL3; THBS1; POSTN; HSPA1A; SPP1; APOC1; FBLN1; CAPG; LGALS1 | 0.006042 |
| GO:0050727 | regulation of inflammatory response | VSIG4; HLA-DRB1; C1QC; C1QB; C1QA; IGHG1; SERPINE1 | 0.006042 |
| GO:0046649 | lymphocyte activation | ITGB1; IGHA1; HLA-A; HLA-DRB1; IGHG1; IGFBP2; SFRP1; LGALS1; VSIG4 | 0.005131 |
| GO:0002697 | regulation of immune effector process | HLA-A; HLA-DRB1; C1QC; C1QB; C1QA; IGHG1; VSIG4 | 0.005131 |
| GO:0060284 | regulation of cell development | SFRP2; SFRP1; DPYSL3; FN1; NBL1; POSTN; SPP1; FBLN1; LGALS1; TIMP2 | 0.006309 |
| GO:0016045 | detection of bacterium | HLA-A; HLA-DRB1 | 0.005383 |
| GO:0048534 | hematopoietic or lymphoid organ development | SFRP2; ITGB1; SFRP1; HLA-DRB1; C1QC; THBS1; HSPA1A; TMEM176B; LGALS1; MYL9 | 0.005383 |
| GO:0007159 | leukocyte cell-cell adhesion | ITGB1; HLA-A; HLA-DRB1; IGFBP2; LGALS1; VSIG4 | 0.005383 |
| GO:0031670 | cellular response to nutrient | POSTN; SFRP1; COL1A1 | 0.005684 |
| GO:0031667 | response to nutrient levels | SFRP2; SFRP1; IGFBP2; POSTN; SPARC; SPP1; COL1A1 | 0.005723 |
| GO:0030111 | regulation of Wnt signaling pathway | SFRP2; SFRP1; SFRP4; IGFBP2; COL1A1; CTHRC1 | 0.007146 |
| GO:0098641 | cadherin binding involved in cell-cell adhesion | S100A11; CNN3 | 0.006023 |
| GO:0016032 | viral process | ITGB1; AP2S1; ITGB5; HLA-A; HLA-DRB1; C1QA; HSPA1A; KRT7; FBLN1; LGALS1 | 0.005855 |
| GO:0001738 | morphogenesis of a polarized epithelium | SFRP2; AP2S1; SFRP1; CTHRC1 | 0.007285 |
| GO:0051246 | regulation of protein metabolic process | SFRP2; TXN; SFRP1; TIMP3; CCN2; FN1; CCL18; PLAUR; VSIG4; HLA-DRB1; C1QC; C1QB; C1QA; IGHG1; IGFBP3; HSPA1A; FBLN1; THBS1; SERPINE1; TIMP2 | 0.007285 |
| GO:0002429 | immune response-activating cell surface receptor signaling pathway | HLA-DRB1; HLA-A; IGHA1; FCGR3A; IGHG1; HLA-DRB5; HLA-DQA1 | 0.006022 |
| GO:0043086 | negative regulation of catalytic activity | SFRP2; SFRP1; TIMP3; TIMP2; CNN3; PLAUR; THBS1; APOC1; SERPINE1 | 0.006022 |
| GO:0002483 | antigen processing and presentation of endogenous peptide antigen | HLA-A; HLA-DRB1 | 0.006322 |
| GO:0032026 | response to magnesium ion | CD14; THBS1 | 0.007671 |
| GO:0002548 | monocyte chemotaxis | CCL18; NBL1; SERPINE1 | 0.00638 |
| GO:0005802 | trans-Golgi network | TGFBI; HLA-DRB1; HLA-DQA1; POSTN; HLA-DRB5 | 0.006418 |
| GO:0031960 | response to corticosteroid | IGFBP2; SPARC; CCN2; COL1A1 | 0.008089 |
| GO:0048678 | response to axon injury | LGALS1; SPP1; DPYSL3 | 0.00685 |
| GO:0071682 | endocytic vesicle lumen | SPARC; HBA2 | 0.006418 |
| GO:0016055 | Wnt signaling pathway | SFRP2; AP2S1; SFRP1; SFRP4; IGFBP2; COL1A1; CTHRC1 | 0.008368 |
| GO:0030176 | integral component of endoplasmic reticulum membrane | HLA-A; HLA-DRB1; HLA-DQA1; HLA-DRB5 | 0.007952 |
| GO:0032677 | regulation of interleukin-8 production | CD14; HSPA1A; SERPINE1 | 0.00932 |
| GO:1903036 | positive regulation of response to wounding | ITGB1; SERPINE1; THBS1 | 0.00932 |
| GO:0043567 | regulation of insulin-like growth factor receptor signaling pathway | IGFBP2; IGFBP3 | 0.009623 |
| GO:0002768 | immune response-regulating cell surface receptor signaling pathway | HLA-DRB1; HLA-A; IGHA1; FCGR3A; IGHG1; HLA-DRB5; HLA-DQA1 | 0.008565 |
| GO:0022409 | positive regulation of cell-cell adhesion | IGFBP2; PLAUR; HLA-A; HLA-DRB1; LGALS1 | 0.010119 |
| GO:0030193 | regulation of blood coagulation | PLAUR; SERPINE1; THBS1 | 0.009059 |
| GO:0060485 | mesenchyme development | ACTA2; SFRP2; SFRP1; FN1; COL1A1 | 0.009374 |
| GO:0001558 | regulation of cell growth | SFRP2; SFRP1; FN1; IGFBP3; HSPA1A; SPP1 | 0.009407 |
| GO:1902105 | regulation of leukocyte differentiation | SFRP1; LGALS1; HLA-DRB1; C1QC; TMEM176B | 0.010617 |
| GO:0001843 | neural tube closure | SFRP2; SFRP1; CTHRC1 | 0.010617 |
| GO:0045992 | negative regulation of embryonic development | SFRP2; COL5A1 | 0.010617 |
| GO:0003094 | glomerular filtration | IGHA1; JCHAIN | 0.010617 |
| GO:0033688 | regulation of osteoblast proliferation | SFRP1; CTHRC1 | 0.010617 |
| GO:0030133 | transport vesicle | DPYSL3; HLA-A; HLA-DRB1; BGN; HLA-DRB5; HLA-DQA1 | 0.00829 |
| GO:0060606 | tube closure | SFRP2; SFRP1; CTHRC1 | 0.009802 |
| GO:0050818 | regulation of coagulation | PLAUR; SERPINE1; THBS1 | 0.010056 |
| GO:0098581 | detection of external biotic stimulus | HLA-A; HLA-DRB1 | 0.010097 |
| GO:0097205 | renal filtration | IGHA1; JCHAIN | 0.010097 |
| GO:0000323 | lytic vacuole | AP2S1; LUM; CTSB; HLA-DRB1; BGN; HLA-DRB5; COL6A1; HLA-DQA1 | 0.00829 |
| GO:0030099 | myeloid cell differentiation | SFRP1; HLA-DRB1; C1QC; THBS1; HSPA1A; MYL9 | 0.010564 |
| GO:0051051 | negative regulation of transport | TXN; SFRP1; SFRP4; THBS1; HSPA1A; APOC1 | 0.010857 |
| GO:0005925 | focal adhesion | ITGB1; ITGB5; CNN3; PLAUR; FHL2; HSPA1A | 0.01176 |
| GO:0043085 | positive regulation of catalytic activity | SFRP2; TXN; SFRP1; CCN2; FN1; CCL18; HLA-DRB1; THBS1; HSPA1A; ITGB1; FBLN1; TIMP2 | 0.010959 |
| GO:0044440 | endosomal part | AP2S1; HLA-A; HLA-DRB1; HLA-DRB5; CD14; CTSB; HLA-DQA1 | 0.01176 |
| GO:1905330 | regulation of morphogenesis of an epithelium | SFRP2; AP2S1; SFRP1; CTHRC1 | 0.012853 |
| GO:0005798 | Golgi-associated vesicle | HLA-A; HLA-DRB1; HLA-DQA1; HLA-DRB5 | 0.00829 |
| GO:0060349 | bone morphogenesis | SFRP2; SFRP4; COL1A1 | 0.011241 |
| GO:0030308 | negative regulation of cell growth | SFRP2; HSPA1A; SFRP1; SPP1 | 0.012941 |
| GO:0002366 | leukocyte activation involved in immune response | TIMP2; PLAUR; CTSB; HLA-DRB1; HSPA1A; CD14; S100A11; LGALS1 | 0.011241 |
| GO:0003401 | axis elongation | SFRP2; SFRP1 | 0.011241 |
| GO:0034110 | regulation of homotypic cell-cell adhesion | PLAUR; LGALS1 | 0.012941 |
| GO:0022407 | regulation of cell-cell adhesion | PLAUR; HLA-A; HLA-DRB1; IGFBP2; LGALS1; VSIG4 | 0.011335 |
| GO:0051174 | regulation of phosphorus metabolic process | SFRP2; TXN; SFRP1; TIMP3; CCN2; FN1; CCL18; PLAUR; HLA-DRB1; THBS1; IGFBP3; APOC1; FBLN1; TIMP2 | 0.01315 |
| GO:1904018 | positive regulation of vasculature development | ITGB1; SFRP2; SERPINE1; THBS1 | 0.01315 |
| GO:0034987 | immunoglobulin receptor binding | IGHA1; IGHG1; JCHAIN | 0.012445 |
| GO:0010717 | regulation of epithelial to mesenchymal transition | SFRP2; SFRP1; COL1A1 | 0.01315 |
| GO:0030135 | coated vesicle | HLA-DRB5; AP2S1; HLA-DRB1; HLA-A; HLA-DQA1 | 0.00829 |
| GO:0005794 | Golgi apparatus | LUM; CCN2; HLA-A; HLA-DRB1; CALU; BGN; HLA-DRB5; POSTN; CD14; SPP1; COL1A1; TGFBI; HLA-DQA1 | 0.01276 |
| GO:0032588 | trans-Golgi network membrane | HLA-DRB1; HLA-DQA1; HLA-DRB5 | 0.00829 |
| GO:0085029 | extracellular matrix assembly | COL1A2; FKBP10 | 0.012572 |
| GO:1900048 | positive regulation of hemostasis | SERPINE1; THBS1 | 0.012572 |
| GO:0048333 | mesodermal cell differentiation | SFRP2; ITGB1 | 0.012572 |
| GO:0030194 | positive regulation of blood coagulation | SERPINE1; THBS1 | 0.014238 |
| GO:0043202 | lysosomal lumen | CTSB; LUM; BGN | 0.00829 |
| GO:0060348 | bone development | SFRP2; SFRP4; BGN; COL1A1 | 0.012957 |
| GO:0061135 | endopeptidase regulator activity | SFRP2; TIMP3; SERPINE1; TIMP2 | 0.012445 |
| GO:0042116 | macrophage activation | VSIG4; C1QA; THBS1 | 0.013124 |
| GO:0003779 | actin binding | ITGB1; CNN3; TPM2; CAPG; CALD1; TAGLN | 0.012445 |
| GO:0050820 | positive regulation of coagulation | SERPINE1; THBS1 | 0.015053 |
| GO:0071229 | cellular response to acid chemical | COL6A1; SFRP1; COL1A2; COL1A1 | 0.013736 |
| GO:0030027 | lamellipodium | ACTA2; ITGB1; CAPG; DPYSL3 | 0.014282 |
| GO:0007044 | cell-substrate junction assembly | SFRP1; FN1; THBS1 | 0.01605 |
| GO:1903037 | regulation of leukocyte cell-cell adhesion | IGFBP2; HLA-A; HLA-DRB1; LGALS1; VSIG4 | 0.016171 |
| GO:0010165 | response to X-ray | SFRP2; SFRP1 | 0.016421 |
| GO:0048640 | negative regulation of developmental growth | SFRP2; SFRP1; SPP1 | 0.016421 |
| GO:0048666 | neuron development | SFRP2; SFRP1; DPYSL3; FN1; NBL1; C1QA; POSTN; SPP1; LGALS1; CTHRC1 | 0.01493 |
| GO:0002062 | chondrocyte differentiation | SFRP2; TGFBI; CCN2 | 0.015086 |
| GO:0030855 | epithelial cell differentiation | ACTA2; CNN3; SFRP4; CTSB; FSTL1; KRT7; SERPINE1; TAGLN | 0.015086 |
| GO:1901701 | cellular response to oxygen-containing compound | TXN; SFRP1; HLA-DRB1; CD14; COL1A2; COL1A1; COL6A1; SPP1; LGALS1; SERPINE1 | 0.015086 |
| GO:0033280 | response to vitamin D | SFRP1; SPP1 | 0.01527 |
| GO:1905114 | cell surface receptor signaling pathway involved in cell-cell signaling | SFRP2; AP2S1; SFRP1; SFRP4; IGFBP2; COL1A1; CTHRC1 | 0.01527 |
| GO:0051171 | regulation of nitrogen compound metabolic process | ACTA2; TIMP3; TIMP2; CCN2; PLAUR; VSIG4; IGHG1; HSPA1A; AEBP1; APOC1; S100A11; FBLN1; THBS1; SERPINE1; TXN; NBL1; LUM; FHL2; FSTL1; HLA-DRB1; IGFBP3; SFRP2; SFRP1; SFRP4; FN1; CCL18; MAGED1; C1QC; C1QB; C1QA; SPP1; COL1A1 | 0.01527 |
| GO:0044267 | cellular protein metabolic process | TIMP3; TIMP2; CCN2; PLAUR; HLA-A; IGHA1; BGN; HSPA1A; FBLN1; FSTL1; SERPINE1; TXN; AP2S1; FKBP10; THBS1; VSIG4; CTSB; HLA-DRB1; IGFBP2; IGFBP3; LGALS1; SFRP2; SFRP1; FN1; CCL18; CALU; SPP1; PRSS23; TGFBI | 0.015284 |
| GO:0051345 | positive regulation of hydrolase activity | ITGB1; SFRP2; SFRP1; CCN2; FN1; CCL18; HSPA1A; FBLN1 | 0.017887 |
| GO:0032649 | regulation of interferon-gamma production | CD14; HLA-DRB1; HLA-A | 0.017887 |
| GO:0030659 | cytoplasmic vesicle membrane | AP2S1; PLAUR; HLA-A; HLA-DRB1; SPARC; HLA-DRB5; CD14; HLA-DQA1 | 0.016604 |
| GO:0034446 | substrate adhesion-dependent cell spreading | POSTN; FBLN1; FN1 | 0.016508 |
| GO:0050678 | regulation of epithelial cell proliferation | SFRP2; MAGED1; SPARC; SFRP1; THBS1 | 0.018802 |
| GO:0042113 | B cell activation | ITGB1; SFRP1; LGALS1; IGHA1; IGHG1 | 0.017084 |
| GO:0009893 | positive regulation of metabolic process | ACTA2; LUM; TIMP2; CCN2; PLAUR; IGHA1; HSPA1A; APOC1; FBLN1; SERPINE1; TXN; THBS1; HLA-DRB1; IGFBP3; JCHAIN; SFRP2; SFRP1; FN1; SFRP4; CCL18; SPP1; COL1A1 | 0.017084 |
| GO:1904019 | epithelial cell apoptotic process | SERPINE1; SFRP4; THBS1 | 0.019408 |
| GO:0097718 | disordered domain specific binding | HSPA1A; FN1 | 0.01647 |
| GO:0005773 | vacuole | AP2S1; LUM; CTSB; HLA-DRB1; BGN; HLA-DRB5; COL6A1; HLA-DQA1 | 0.01822 |
| GO:0031175 | neuron projection development | SFRP2; SFRP1; DPYSL3; FN1; NBL1; POSTN; SPP1; LGALS1; CTHRC1 | 0.020502 |
| GO:0050953 | sensory perception of light stimulus | TGFBI; TIMP3; LUM; COL1A1 | 0.020502 |
| GO:0019058 | viral life cycle | ITGB1; HSPA1A; HLA-DRB1; ITGB5; LGALS1 | 0.020502 |
| GO:0043312 | neutrophil degranulation | TIMP2; PLAUR; CTSB; HSPA1A; CD14; S100A11 | 0.018919 |
| GO:0001838 | embryonic epithelial tube formation | SFRP2; SFRP1; CTHRC1 | 0.020502 |
| GO:0045667 | regulation of osteoblast differentiation | SFRP2; SFRP1; CTHRC1 | 0.020502 |
| GO:0001914 | regulation of T cell mediated cytotoxicity | HLA-A; HLA-DRB1 | 0.020502 |
| GO:0071542 | dopaminergic neuron differentiation | SFRP2; SFRP1 | 0.020502 |
| GO:0005109 | frizzled binding | SFRP1; CTHRC1 | 0.020502 |
| GO:0030182 | neuron differentiation | SFRP2; SFRP1; DPYSL3; FN1; NBL1; C1QA; POSTN; SPP1; LGALS1; CTHRC1; TIMP2 | 0.019529 |
| GO:0048762 | mesenchymal cell differentiation | SFRP2; SFRP1; FN1; COL1A1 | 0.019582 |
| GO:0010038 | response to metal ion | IGFBP2; CD14; SPARC; C1QA; THBS1 | 0.019856 |
| GO:0010470 | regulation of gastrulation | SFRP2; COL5A1 | 0.021359 |
| GO:0030016 | myofibril | FHL2; CALD1; TPM2; MYL9 | 0.013816 |
| GO:0002446 | neutrophil mediated immunity | TIMP2; PLAUR; CTSB; HSPA1A; CD14; S100A11 | 0.022363 |
| GO:0002688 | regulation of leukocyte chemotaxis | NBL1; SERPINE1; THBS1 | 0.022363 |
| GO:0032368 | regulation of lipid transport | THBS1; SPP1; APOC1 | 0.022665 |
| GO:0010712 | regulation of collagen metabolic process | ITGB1; CCN2 | 0.021612 |
| GO:0033574 | response to testosterone | SPP1; THBS1 | 0.021612 |
| GO:0001894 | tissue homeostasis | IGHA1; SPP1; CCN2; JCHAIN | 0.022749 |
| GO:0036230 | granulocyte activation | TIMP2; PLAUR; CTSB; HSPA1A; CD14; S100A11 | 0.021694 |
| GO:0042742 | defense response to bacterium | SERPINE1; HLA-A; IGHA1; IGHG1; JCHAIN | 0.021694 |
| GO:0042476 | odontogenesis | COL1A2; SERPINE1; COL1A1 | 0.021966 |
| GO:1902903 | regulation of supramolecular fiber organization | SFRP1; HSPA1A; CAPG; AEBP1; CCN2 | 0.023358 |

**Table S3.** GO terms enriched by the significantly upregulated genes in responders.

| **GO Term** | **Term name** | **List of upregulated genes** | **Q-value** |
| --- | --- | --- | --- |
| GO:0042113 | B cell activation | CR2; SYVN1; CD22; CD40; CD19; TCIRG1; PTPN6; SASH3; BLK; TBC1D10C; FCRL1; BANK1; MS4A1; CD79B; IGHM; CD79A | 1.59E-14 |
| GO:0046649 | lymphocyte activation | CR2; SYVN1; IL4R; CD22; RIPOR2; CD40; CD19; TCIRG1; PTPN6; SASH3; BLK; TBC1D10C; FCRL1; BANK1; MS4A1; CD79B; IGHM; CD79A | 1.18E-11 |
| GO:0050778 | positive regulation of immune response | CR2; PAX5; IL4R; CD22; CFD; CD40; CD36; PTPN6; CD79A; IGLV3-1; LTF; BLK; CLU; SASH3; CD19; CD79B; IGHM; MS4A1 | 5.20E-10 |
| GO:0002684 | positive regulation of immune system process | CR2; PAX5; RIPOR2; IL4R; CD22; CFD; CD40; CD36; PTPN6; CD79A; SASH3; IGLV3-1; LTF; BLK; CLU; CXCL13; CD19; CD79B; IGHM; MS4A1 | 2.57E-10 |
| GO:0048584 | positive regulation of response to stimulus | CD36; FABP4; MS4A1; SEMA4D; IL4R; CD79B; IGHM; BANK1; CD79A; G0S2; TRAF5; CD22; CD40; PAX5; LTF; BLK; CLU; CXCL13; PTPN6; CR2; CFD; ABCA7; IGLV3-1; SASH3; RIPOR2; CD19 | 4.30E-10 |
| GO:0050776 | regulation of immune response | CR2; PAX5; IL4R; CD22; CFD; CD40; CD36; PTPN6; CD79A; SASH3; IGLV3-1; LTF; BLK; CLU; CXCL13; CD19; CD79B; IGHM; MS4A1 | 5.11E-10 |
| GO:0002460 | adaptive immune response based on somatic recombination of immune receptors built from immunoglobulin superfamily domains | CR2; IL4R; CD40; PTPN6; TCIRG1; SASH3; IGLV3-1; CLU; CXCL13; IGHM; CD19 | 8.16E-08 |
| GO:0002521 | leukocyte differentiation | CR2; SYVN1; IL4R; CD19; TCIRG1; LTF; BLK; SASH3; MS4A1; CD79B; PTPN6; CD79A | 1.27E-07 |
| GO:0002768 | immune response-regulating cell surface receptor signaling pathway | CR2; CD22; CD40; CD19; PAX5; PTPN6; IGLV3-1; BLK; MS4A1; CD79B; IGHM; CD79A | 1.38E-07 |
| GO:0019724 | B cell mediated immunity | CR2; IL4R; CD40; PTPN6; TCIRG1; IGLV3-1; CLU; IGHM; CD19 | 6.88E-07 |
| GO:0030098 | lymphocyte differentiation | CR2; SYVN1; IL4R; CD19; TCIRG1; SASH3; MS4A1; CD79B; PTPN6; CD79A | 3.65E-07 |
| GO:0002697 | regulation of immune effector process | CR2; IL4R; CD22; CD40; CD36; SASH3; IGLV3-1; BLK; CLU; PTPN6; CD19 | 4.31E-07 |
| GO:0002449 | lymphocyte mediated immunity | CR2; IL4R; CD40; PTPN6; TCIRG1; IGLV3-1; CLU; SASH3; IGHM; CD19 | 4.95E-07 |
| GO:0046651 | lymphocyte proliferation | CR2; CD22; CD40; CD19; BLK; SASH3; MS4A1; PTPN6; CD79A | 4.95E-07 |
| GO:0002429 | immune response-activating cell surface receptor signaling pathway | CR2; CD22; CD19; PAX5; PTPN6; IGLV3-1; BLK; MS4A1; CD79B; IGHM; CD79A | 4.95E-07 |
| GO:0051249 | regulation of lymphocyte activation | IL4R; CD22; RIPOR2; CD40; PTPN6; SASH3; BLK; BANK1; TBC1D10C; IGHM; CD19 | 2.50E-06 |
| GO:0050851 | antigen receptor-mediated signaling pathway | CD22; CD19; PAX5; PTPN6; BLK; MS4A1; CD79B; IGHM; CD79A | 5.01E-06 |
| GO:0002694 | regulation of leukocyte activation | IL4R; CD22; RIPOR2; CD40; PTPN6; SASH3; BLK; BANK1; TBC1D10C; IGHM; CD19 | 3.28E-06 |
| GO:0050865 | regulation of cell activation | IL4R; CD22; RIPOR2; CD40; PTPN6; SASH3; BLK; BANK1; TBC1D10C; IGHM; CD19 | 5.57E-06 |
| GO:0050854 | regulation of antigen receptor-mediated signaling pathway | PAX5; CD22; PTPN6; BLK; CD19 | 1.79E-05 |
| GO:0030097 | hemopoiesis | CR2; SYVN1; IL4R; CD19; TCIRG1; LTF; BLK; SASH3; MS4A1; CD79B; PTPN6; CD79A | 3.63E-05 |
| GO:0002920 | regulation of humoral immune response | CR2; IGLV3-1; CLU; CXCL13; PTPN6; CD19 | 3.85E-05 |
| GO:0048534 | hematopoietic or lymphoid organ development | CR2; SYVN1; IL4R; CD19; TCIRG1; LTF; BLK; SASH3; MS4A1; CD79B; PTPN6; CD79A | 2.95E-05 |
| GO:0002695 | negative regulation of leukocyte activation | IL4R; RIPOR2; BLK; BANK1; TBC1D10C; PTPN6 | 0.000111 |
| GO:0006954 | inflammatory response | CR2; IL4R; CIITA; CD40; CD36; TCIRG1; FABP4; IGLV3-1; CLU; CXCL13; CD19 | 7.38E-05 |
| GO:0050866 | negative regulation of cell activation | IL4R; RIPOR2; BLK; BANK1; TBC1D10C; PTPN6 | 0.000168 |
| GO:0002366 | leukocyte activation involved in immune response | IL4R; CFD; CD40; CD36; TCIRG1; LTF; BLK; TBC1D10C; PTPN6; CD19 | 0.00012 |
| GO:0032101 | regulation of response to external stimulus | CR2; RIPOR2; CD36; FABP4; IGLV3-1; LTF; BLK; CLU; CXCL13; SEMA4D; CD19 | 0.000151 |
| GO:0002683 | negative regulation of immune system process | IL4R; CD22; RIPOR2; LTF; BLK; BANK1; TBC1D10C; PTPN6 | 0.000216 |
| GO:0048878 | chemical homeostasis | EHD1; ATP2A3; TMC8; CD40; CD36; TCIRG1; FABP4; LTF; CXCL13; CD19; PTPN6; MS4A1 | 0.00023 |
| GO:0050801 | ion homeostasis | ATP2A3; TMC8; CD40; CD36; TCIRG1; LTF; CXCL13; CD19; PTPN6; MS4A1 | 0.000416 |
| GO:0030003 | cellular cation homeostasis | ATP2A3; CD40; CD36; TCIRG1; LTF; CXCL13; CD19; PTPN6; MS4A1 | 0.000416 |
| GO:0032640 | tumor necrosis factor production | CLU; SASH3; LTF; CD36; PTPN6 | 0.000306 |
| GO:0002274 | myeloid leukocyte activation | IL4R; CFD; CD36; TCIRG1; LTF; BLK; CLU; TBC1D10C; PTPN6 | 0.000306 |
| GO:1902531 | regulation of intracellular signal transduction | TRAF5; CD22; RIPOR2; CD40; CD36; ARHGEF1; SYVN1; ABCA7; LTF; CLU; BANK1; TBC1D10C; SEMA4D; PTPN6; CD19 | 0.000416 |
| GO:0010941 | regulation of cell death | G0S2; TRAF5; ATP2A3; TMC8; FCMR; CD40; CD36; ARHGEF1; SYVN1; LTF; BLK; CLU; SEMA4D; PTPN6 | 0.000306 |
| GO:0019730 | antimicrobial humoral response | CLU; CXCL13; ITLN1; IGHM; LTF | 0.000306 |
| GO:1903555 | regulation of tumor necrosis factor superfamily cytokine production | CLU; SASH3; LTF; CD36; PTPN6 | 0.000416 |
| GO:0002700 | regulation of production of molecular mediator of immune response | CD22; SASH3; CD40; CD36; IL4R | 0.000416 |
| GO:0006873 | cellular ion homeostasis | ATP2A3; CD40; CD36; TCIRG1; LTF; CXCL13; CD19; PTPN6; MS4A1 | 0.000306 |
| GO:0060142 | regulation of syncytium formation by plasma membrane fusion | RIPOR2; IL4R; EHD1 | 0.000453 |
| GO:0051092 | positive regulation of NF-kappaB transcription factor activity | CLU; TRAF5; CD40; CD36; LTF | 0.000562 |
| GO:0043067 | regulation of programmed cell death | G0S2; TRAF5; ATP2A3; TMC8; FCMR; CD40; ARHGEF1; SYVN1; LTF; BLK; CLU; SEMA4D; PTPN6 | 0.000619 |
| GO:0002275 | myeloid cell activation involved in immune response | IL4R; CFD; CD36; TCIRG1; LTF; BLK; TBC1D10C; PTPN6 | 0.000528 |
| GO:0002444 | myeloid leukocyte mediated immunity | IL4R; CFD; CD36; TCIRG1; LTF; BLK; TBC1D10C; PTPN6 | 0.000528 |
| GO:0009966 | regulation of signal transduction | SYVN1; G0S2; TRAF5; CD22; RIPOR2; TMC8; CD40; CD36; ARHGEF1; PAX5; ABCA7; LTF; BLK; CLU; BANK1; TBC1D10C; SEMA4D; PTPN6; CD19 | 0.000563 |
| GO:0002455 | humoral immune response mediated by circulating immunoglobulin | CR2; CLU; IGLV3-1; IGHM; PTPN6 | 0.000592 |
| GO:0010646 | regulation of cell communication | TCIRG1; G0S2; TRAF5; SYVN1; CD22; RIPOR2; TMC8; CD40; CD36; ARHGEF1; PAX5; ABCA7; LTF; BLK; CLU; BANK1; TBC1D10C; SEMA4D; PTPN6; CD19 | 0.000728 |
| GO:0120162 | positive regulation of cold-induced thermogenesis | G0S2; FABP4; IL4R; CD36 | 0.000784 |
| GO:0045055 | regulated exocytosis | IL4R; CFD; CD36; TCIRG1; LTF; BLK; CLU; TBC1D10C; PTPN6 | 0.000851 |
| GO:0048522 | positive regulation of cellular process | IGHM; EHD1; ATP2A3; CD36; SEMA4D; TRAF5; RERE; CIITA; ARHGEF1; BANK1; G0S2; IL4R; CD40; SPIB; LTF; BLK; CLU; CXCL13; PTPN6; PAX5; RIPOR2; TCIRG1; ABCA7; ITLN1; SASH3; CD19 | 0.000854 |
| GO:0080134 | regulation of response to stress | CR2; TRAF5; CD40; CD36; SYVN1; ABCA7; FABP4; IGLV3-1; LTF; BLK; CLU; PTPN6; CD19 | 0.000894 |
| GO:0150094 | amyloid-beta clearance by cellular catabolic process | ABCA7; CD36 | 0.000912 |
| GO:0010886 | positive regulation of cholesterol storage | EHD1; CD36 | 0.001337 |
| GO:0070062 | extracellular exosome | CR2; EHD1; CD22; CFD; TMC8; CD40; PTPN6; FABP4; ITLN1; LTF; CLU; CD19; CD79B; IGHM; MS4A1 | 0.00289 |
| GO:0007520 | myoblast fusion | RIPOR2; IL4R; EHD1 | 0.000993 |
| GO:0006915 | apoptotic process | SYVN1; G0S2; TRAF5; ATP2A3; TMC8; FCMR; CD40; ARHGEF1; TCIRG1; LTF; BLK; CLU; SEMA4D; PTPN6 | 0.000993 |
| GO:0002703 | regulation of leukocyte mediated immunity | PTPN6; SASH3; CD40; BLK; IL4R | 0.001586 |
| GO:0002699 | positive regulation of immune effector process | IL4R; SASH3; CD36; BLK; CD40 | 0.001998 |
| GO:0031095 | platelet dense tubular network membrane | EHD1; ATP2A3 | 0.00289 |
| GO:0030449 | regulation of complement activation | CR2; CLU; IGLV3-1; CD19 | 0.002057 |
| GO:2000257 | regulation of protein activation cascade | CR2; CLU; IGLV3-1; CD19 | 0.001559 |
| GO:0071345 | cellular response to cytokine stimulus | IL4R; CIITA; RIPOR2; CD40; CD36; TCIRG1; FABP4; CXCL13; PTPN6; TRAF5 | 0.002198 |
| GO:0051246 | regulation of protein metabolic process | CR2; TRAF5; ATP2A3; TBC1D10C; CD40; CD36; PAX5; ABCA7; FABP4; ITLN1; IGLV3-1; LTF; CLU; BANK1; SEMA4D; PTPN6; CD19 | 0.002198 |
| GO:0070663 | regulation of leukocyte proliferation | CD22; SASH3; CD40; BLK; PTPN6 | 0.002198 |
| GO:0060627 | regulation of vesicle-mediated transport | IL4R; EHD1; CD22; CD36; ABCA7; BLK; CLU | 0.001915 |
| GO:0031347 | regulation of defense response | CR2; CD40; CD36; FABP4; IGLV3-1; LTF; CLU; PTPN6; CD19 | 0.002198 |
| GO:0001817 | regulation of cytokine production | IL4R; CD40; CD36; SASH3; LTF; CLU; BANK1; PTPN6 | 0.001915 |
| GO:1903573 | negative regulation of response to endoplasmic reticulum stress | SYVN1; CLU; ABCA7 | 0.002198 |
| GO:0051174 | regulation of phosphorus metabolic process | TRAF5; TBC1D10C; CD40; CD36; ABCA7; FABP4; ITLN1; LTF; CLU; BANK1; SEMA4D; PTPN6; CD19 | 0.002198 |
| GO:0071310 | cellular response to organic substance | SYVN1; IL4R; EHD1; CIITA; RIPOR2; BCL11A; CD40; CD36; TCIRG1; ABCA7; FABP4; LTF; CLU; CXCL13; PTPN6; TRAF5 | 0.00199 |
| GO:0031094 | platelet dense tubular network | EHD1; ATP2A3 | 0.00289 |
| GO:0035631 | CD40 receptor complex | CD40; TRAF5 | 0.00289 |
| GO:0016310 | phosphorylation | TRAF5; CIITA; CD40; CD36; ABCA7; FABP4; ITLN1; LTF; BLK; CLU; BANK1; TBC1D10C; SEMA4D; PTPN6; CD19 | 0.002929 |
| GO:0120161 | regulation of cold-induced thermogenesis | G0S2; FABP4; IL4R; CD36 | 0.002621 |
| GO:0048585 | negative regulation of response to stimulus | IL4R; CD22; RIPOR2; TBC1D10C; SYVN1; ABCA7; CXCL13; LTF; CLU; BANK1; SEMA4D; PTPN6 | 0.002621 |
| GO:0034097 | response to cytokine | IL4R; CIITA; RIPOR2; CD40; CD36; TCIRG1; FABP4; CXCL13; PTPN6; TRAF5 | 0.002664 |
| GO:0051091 | positive regulation of DNA-binding transcription factor activity | CLU; TRAF5; CD40; CD36; LTF | 0.002778 |
| GO:0016192 | vesicle-mediated transport | IL4R; EHD1; CD22; CFD; CD36; PTPN6; TCIRG1; ABCA7; IGLV3-1; LTF; BLK; CLU; TBC1D10C; IGHM | 0.002866 |
| GO:0005887 | integral component of plasma membrane | SEMA4D; IL4R; CD22; TMC8; CD40; CD36; TCIRG1; FCRL1; TRAF5; CD19; CD79B; MS4A1 | 0.003809 |
| GO:0022407 | regulation of cell-cell adhesion | IL4R; RIPOR2; PTPN6; BLK; CXCL13; SASH3 | 0.003284 |
| GO:0009893 | positive regulation of metabolic process | RERE; SPIB; G0S2; SEMA4D; IL4R; ATP2A3; CIITA; BCL11A; CD40; CD36; PAX5; ABCA7; FABP4; ITLN1; LTF; CLU; BANK1; TRAF5; CD19 | 0.003284 |
| GO:0006958 | complement activation, classical pathway | CR2; CLU; IGLV3-1; IGHM | 0.003321 |
| GO:0010885 | regulation of cholesterol storage | EHD1; CD36 | 0.003556 |
| GO:0006957 | complement activation, alternative pathway | CR2; CFD | 0.003556 |
| GO:1905153 | regulation of membrane invagination | ABCA7; CD36 | 0.004675 |
| GO:0002819 | regulation of adaptive immune response | PTPN6; SASH3; CD40; IL4R | 0.005098 |
| GO:1900221 | regulation of amyloid-beta clearance | CLU; ABCA7 | 0.003948 |
| GO:0010878 | cholesterol storage | EHD1; CD36 | 0.004353 |
| GO:0043087 | regulation of GTPase activity | RASGRP2; SEMA4D; CD40; ARHGEF1; CXCL13; TBC1D10C | 0.006824 |
| GO:0048523 | negative regulation of cellular process | TMC8; CD36; FABP4; SEMA4D; RERE; CIITA; BCL11A; SYVN1; BANK1; IL4R; CD22; PAX5; LTF; BLK; CLU; CXCL13; PTPN6; RIPOR2; FCMR; ABCA7; TBC1D10C; CD19 | 0.00567 |
| GO:0043312 | neutrophil degranulation | CFD; CD36; TCIRG1; LTF; TBC1D10C; PTPN6 | 0.00567 |
| GO:0045121 | membrane raft | CD79A; ITLN1; MS4A1; CD36; CD19 | 0.00719 |
| GO:0033630 | positive regulation of cell adhesion mediated by integrin | CXCL13; PTPN6 | 0.007356 |
| GO:0051050 | positive regulation of transport | IL4R; EHD1; CD36; ABCA7; ITLN1; BLK; CLU; CD19 | 0.005818 |
| GO:0070977 | bone maturation | SEMA4D; LTF | 0.007834 |
| GO:0002446 | neutrophil mediated immunity | CFD; CD36; TCIRG1; LTF; TBC1D10C; PTPN6 | 0.007841 |
| GO:0010942 | positive regulation of cell death | G0S2; ATP2A3; CD40; CD36; ARHGEF1; BLK; CLU | 0.007841 |
| GO:0071219 | cellular response to molecule of bacterial origin | CXCL13; CD40; CD36; LTF | 0.006542 |
| GO:0036230 | granulocyte activation | CFD; CD36; TCIRG1; LTF; TBC1D10C; PTPN6 | 0.006542 |
| GO:0060341 | regulation of cellular localization | IL4R; EHD1; RIPOR2; CD36; ABCA7; BLK; PTPN6; CD19 | 0.006703 |
| GO:0042832 | defense response to protozoan | CD40; IL4R | 0.006841 |
| GO:0007599 | hemostasis | EHD1; PTPN6; CD36; BLK; CD40 | 0.006841 |
| GO:0005811 | lipid droplet | FABP4; G0S2; EHD1 | 0.008479 |
| GO:1905954 | positive regulation of lipid localization | ABCA7; EHD1; CD36 | 0.006841 |
| GO:1990000 | amyloid fibril formation | CLU; CD36 | 0.006841 |
| GO:0031664 | regulation of lipopolysaccharide-mediated signaling pathway | LTF; CD36 | 0.006841 |
| GO:0010884 | positive regulation of lipid storage | EHD1; CD36 | 0.006841 |
| GO:0007159 | leukocyte cell-cell adhesion | RIPOR2; SASH3; SEMA4D; IL4R; PTPN6 | 0.006841 |
| GO:0048799 | animal organ maturation | SEMA4D; LTF | 0.007251 |
| GO:0043085 | positive regulation of catalytic activity | RASGRP2; ATP2A3; TBC1D10C; CD40; ARHGEF1; LTF; CLU; CXCL13; SEMA4D; CD19 | 0.007363 |
| GO:0032091 | negative regulation of protein binding | RIPOR2; TMC8; ATP2A3 | 0.010641 |
| GO:0002526 | acute inflammatory response | CR2; CLU; IGLV3-1; CD19 | 0.011663 |
| GO:0050858 | negative regulation of antigen receptor-mediated signaling pathway | CD22; PTPN6 | 0.011827 |
| GO:0051259 | protein complex oligomerization | RIPOR2; ITLN1; EHD1; MS4A1 | 0.011985 |
| GO:0051049 | regulation of transport | IL4R; EHD1; CD22; CD36; TCIRG1; ABCA7; ITLN1; BLK; CLU; PTPN6; CD19 | 0.00943 |
| GO:0051128 | regulation of cellular component organization | RASGRP2; IL4R; EHD1; CD22; BCL11A; TMC8; TBC1D10C; CD36; PAX5; ABCA7; CLU; CXCL13; SEMA4D; RIPOR2 | 0.009983 |
| GO:0015405 | P-P-bond-hydrolysis-driven transmembrane transporter activity | TCIRG1; ABCA7; ATP2A3 | 0.045965 |
| GO:0051345 | positive regulation of hydrolase activity | RASGRP2; ATP2A3; SEMA4D; CD40; ARHGEF1; CXCL13; TBC1D10C | 0.01334 |
| GO:0015399 | primary active transmembrane transporter activity | TCIRG1; ABCA7; ATP2A3 | 0.080393 |
| GO:0098751 | bone cell development | LTF; PTPN6 | 0.010353 |
| GO:0045667 | regulation of osteoblast differentiation | TCIRG1; SEMA4D; LTF | 0.013515 |
| GO:0033006 | regulation of mast cell activation involved in immune response | IL4R; BLK | 0.013829 |
| GO:0019221 | cytokine-mediated signaling pathway | TRAF5; CIITA; CD40; CD36; CXCL13; PTPN6; IL4R | 0.010937 |
| GO:0009968 | negative regulation of signal transduction | CD22; RIPOR2; SYVN1; ABCA7; LTF; CLU; BANK1; TBC1D10C; PTPN6 | 0.01413 |
| GO:0002696 | positive regulation of leukocyte activation | IL4R; SASH3; PTPN6; CD40; IGHM | 0.01413 |
| GO:0010647 | positive regulation of cell communication | G0S2; TRAF5; CD40; CD36; ABCA7; LTF; BLK; BANK1; SEMA4D; PTPN6; CD19 | 0.01413 |
| GO:0045429 | positive regulation of nitric oxide biosynthetic process | CLU; CD36 | 0.01413 |
| GO:0032268 | regulation of cellular protein metabolic process | TRAF5; ATP2A3; TBC1D10C; CD40; CD36; PAX5; ABCA7; FABP4; ITLN1; LTF; CLU; BANK1; SEMA4D; PTPN6 | 0.01413 |
| GO:0023056 | positive regulation of signaling | G0S2; TRAF5; CD40; CD36; ABCA7; LTF; BLK; BANK1; SEMA4D; PTPN6; CD19 | 0.01236 |
| GO:0002698 | negative regulation of immune effector process | CD22; IL4R; PTPN6 | 0.014277 |
| GO:1904407 | positive regulation of nitric oxide metabolic process | CLU; CD36 | 0.012546 |
| GO:0045785 | positive regulation of cell adhesion | CXCL13; SASH3; IL4R; CD36; PTPN6 | 0.012751 |
| GO:0050867 | positive regulation of cell activation | IL4R; SASH3; PTPN6; CD40; IGHM | 0.014645 |
| GO:0022603 | regulation of anatomical structure morphogenesis | IL4R; EHD1; RIPOR2; BCL11A; CD40; CD36; CXCL13; SEMA4D | 0.013919 |
| GO:0098542 | defense response to other organism | IL4R; CD40; CD36; LTF; CXCL13; IGHM | 0.014043 |
| GO:0032675 | regulation of interleukin-6 production | BANK1; PTPN6; CD36 | 0.016333 |
| GO:0022409 | positive regulation of cell-cell adhesion | CXCL13; SASH3; IL4R; PTPN6 | 0.017184 |
| GO:0007162 | negative regulation of cell adhesion | RIPOR2; SEMA4D; IL4R; PTPN6 | 0.015528 |
| GO:0001819 | positive regulation of cytokine production | CLU; SASH3; CD40; CD36; IL4R | 0.017524 |
| GO:0006897 | endocytosis | EHD1; CD22; CD36; ABCA7; IGLV3-1; CLU; IGHM | 0.017524 |
| GO:0042127 | regulation of cell proliferation | IL4R; CD22; CD40; SASH3; TCIRG1; LTF; BLK; CLU; PTPN6; TRAF5 | 0.015936 |
| GO:0007249 | I-kappaB kinase/NF-kappaB signaling | TRAF5; CD40; CD36; LTF | 0.015936 |
| GO:1902105 | regulation of leukocyte differentiation | PTPN6; SASH3; IL4R; LTF | 0.017524 |
| GO:0051172 | negative regulation of nitrogen compound metabolic process | RERE; CIITA; BCL11A; TBC1D10C; CD36; PAX5; ABCA7; FABP4; LTF; CLU; BANK1; SEMA4D; PTPN6 | 0.017751 |
| GO:0010648 | negative regulation of cell communication | CD22; RIPOR2; SYVN1; ABCA7; LTF; CLU; BANK1; TBC1D10C; PTPN6 | 0.017751 |
| GO:0023057 | negative regulation of signaling | CD22; RIPOR2; SYVN1; ABCA7; LTF; CLU; BANK1; TBC1D10C; PTPN6 | 0.016816 |
| GO:0033003 | regulation of mast cell activation | IL4R; BLK | 0.017751 |
| GO:0009967 | positive regulation of signal transduction | G0S2; TRAF5; CD40; CD36; ABCA7; LTF; BANK1; SEMA4D; PTPN6; CD19 | 0.017751 |
| GO:0043300 | regulation of leukocyte degranulation | IL4R; BLK | 0.018712 |
| GO:0099024 | plasma membrane invagination | ABCA7; IGHM; CD36 | 0.018712 |
| GO:0042110 | T cell activation | TCIRG1; RIPOR2; SASH3; IL4R; PTPN6 | 0.018892 |
| GO:0060402 | calcium ion transport into cytosol | MS4A1; PTPN6; CD19 | 0.018909 |
| GO:0050727 | regulation of inflammatory response | CR2; CLU; FABP4; IGLV3-1; CD19 | 0.019178 |
| GO:0034976 | response to endoplasmic reticulum stress | SYVN1; CLU; ABCA7; ATP2A3 | 0.018909 |
| GO:0006811 | ion transport | ATP2A3; TMC8; CD36; TCIRG1; ABCA7; FABP4; LTF; CD19; PTPN6; MS4A1 | 0.019005 |
| GO:0051147 | regulation of muscle cell differentiation | RIPOR2; IL4R; EHD1 | 0.019494 |
| GO:0033628 | regulation of cell adhesion mediated by integrin | CXCL13; PTPN6 | 0.019005 |
| GO:0043303 | mast cell degranulation | IL4R; BLK | 0.019558 |
| GO:0000165 | MAPK cascade | TRAF5; CD40; CD36; ABCA7; BANK1; TBC1D10C; PTPN6 | 0.019678 |
| GO:0010324 | membrane invagination | ABCA7; IGHM; CD36 | 0.019678 |
| GO:0002448 | mast cell mediated immunity | IL4R; BLK | 0.020829 |
| GO:0030139 | endocytic vesicle | TCIRG1; EHD1; LTF; CD36 | 0.026411 |
| GO:1905952 | regulation of lipid localization | ABCA7; EHD1; CD36 | 0.020156 |
| GO:0070371 | ERK1 and ERK2 cascade | ABCA7; TBC1D10C; PTPN6; CD36 | 0.022093 |
| GO:0045428 | regulation of nitric oxide biosynthetic process | CLU; CD36 | 0.020995 |
| GO:0051100 | negative regulation of binding | RIPOR2; TMC8; ATP2A3 | 0.021483 |
| GO:0032496 | response to lipopolysaccharide | CXCL13; CD40; CD36; LTF | 0.021675 |
| GO:1903037 | regulation of leukocyte cell-cell adhesion | RIPOR2; SASH3; IL4R; PTPN6 | 0.022978 |
| GO:0006968 | cellular defense response | TCIRG1; FCMR | 0.021874 |
| GO:0042987 | amyloid precursor protein catabolic process | CLU; ABCA7 | 0.023081 |
| GO:0034774 | secretory granule lumen | CLU; CFD; LTF; PTPN6 | 0.026411 |
| GO:1904724 | tertiary granule lumen | LTF; PTPN6 | 0.03234 |
| GO:0050435 | amyloid-beta metabolic process | CLU; ABCA7 | 0.023558 |
| GO:0051240 | positive regulation of multicellular organismal process | G0S2; IL4R; CD40; CD36; ABCA7; FABP4; LTF; CLU; SEMA4D; SASH3 | 0.02253 |
| GO:0046903 | secretion | IL4R; CFD; CD36; TCIRG1; LTF; BLK; CLU; TBC1D10C; PTPN6 | 0.023652 |
| GO:0065003 | protein-containing complex assembly | EHD1; RIPOR2; TMC8; CD40; CD36; ABCA7; ITLN1; CLU; CXCL13; MS4A1 | 0.023783 |
| GO:0031324 | negative regulation of cellular metabolic process | RERE; CIITA; BCL11A; TBC1D10C; CD36; PAX5; ABCA7; FABP4; LTF; CLU; BANK1; SEMA4D; PTPN6 | 0.025475 |
| GO:0031663 | lipopolysaccharide-mediated signaling pathway | LTF; CD36 | 0.024095 |
| GO:0045597 | positive regulation of cell differentiation | IL4R; EHD1; RIPOR2; CD36; LTF; SEMA4D; SASH3 | 0.025543 |
| GO:0002237 | response to molecule of bacterial origin | CXCL13; CD40; CD36; LTF | 0.024617 |
| GO:0031526 | brush border membrane | ITLN1; CD36 | 0.03234 |
| GO:0032655 | regulation of interleukin-12 production | CD40; CD36 | 0.025818 |
| GO:0022408 | negative regulation of cell-cell adhesion | RIPOR2; IL4R; PTPN6 | 0.026226 |
| GO:0060205 | cytoplasmic vesicle lumen | CLU; CFD; LTF; PTPN6 | 0.03234 |
| GO:0101003 | ficolin-1-rich granule membrane | TCIRG1; TBC1D10C | 0.03234 |

**Table S4**. Spelling of the acronyms used in the main text.

| **Gene acronym** | **Definition** |
| --- | --- |
| RECIST 1.1 | Response evaluation criteria in solid tumours 1.1 |
| FFPE | Fresh-frozen paraffin-embedded |
| FCGR2B | Fc fragment of IgD receptor IIb |
| HFE | Homeostatic iron regulator |
| IL1R1 | Interleukin 1 receptor type 1 |
| HLX | H2.0 like homeobox |
| HLA | Human leukocyte antigen |
| PTPN6 | Protein tyrosine phosphatase non-receptor type 6 |
| RASGRP2 | RAS guanyl releasing protein 2 |
| PDCD1 | Programmed cell death 1 |
| HAVCR2 | Hepatitis A Virus Cellular Receptor 2 |
| LAG-3 | Lymphocyte Activation Gene-3 |
| TIGIT | T Cell Immunoreceptor with Ig And ITIM Domains |
| BTLA | B- and T-lymphocyte attenuator |
| B3GAT1 | Beta-1,3-Glucuronyltransferase 1 |
| KLRG-1 | Killer Cell Lectin Like Receptor G1 |
| TIM-3 | T cell immunoglobulin domain and mucin domain 3 |
| PTCH1 | Patched1 |
